# Genome-wide association study reveals *WRKY42* as a novel plant transcription factor that influences oviposition preference of *Pieris* butterflies

**DOI:** 10.1093/jxb/erac501

**Published:** 2022-12-23

**Authors:** Silvia Coolen, Marcel Van Dijen, Johan A Van Pelt, Joop J A Van Loon, Corné M J Pieterse, Saskia C M Van Wees

**Affiliations:** Plant-Microbe Interactions, Department of Biology, Utrecht University, P.O. Box 800.56, 3508 TB, Utrecht, The Netherlands; Microbiology, Radboud Institute for Biological and Environmental Sciences (RIBES), Radboud University, P.O. Box 9010, 6500 GL, Nijmegen, The Netherlands; Plant-Microbe Interactions, Department of Biology, Utrecht University, P.O. Box 800.56, 3508 TB, Utrecht, The Netherlands; Plant-Microbe Interactions, Department of Biology, Utrecht University, P.O. Box 800.56, 3508 TB, Utrecht, The Netherlands; Laboratory of Entomology, Wageningen University, P.O. Box 16, 6700 AA, Wageningen, The Netherlands; Plant-Microbe Interactions, Department of Biology, Utrecht University, P.O. Box 800.56, 3508 TB, Utrecht, The Netherlands; Plant-Microbe Interactions, Department of Biology, Utrecht University, P.O. Box 800.56, 3508 TB, Utrecht, The Netherlands; University of Birmingham, UK

**Keywords:** *AOC1*, *Arabidopsis thaliana*, butterfly, caterpillar performance, GWAS, HapMap, host-plant selection, oviposition preference, *Pieris*, *WKRY42*

## Abstract

Insect herbivores are amongst the most destructive plant pests, damaging both naturally occurring and domesticated plants. As sessile organisms, plants make use of structural and chemical barriers to counteract herbivores. However, over 75% of herbivorous insect species are well adapted to their host’s defenses and these specialists are generally difficult to ward off. By actively antagonizing the number of insect eggs deposited on plants, future damage by the herbivore’s offspring can be limited. Therefore, it is important to understand which plant traits influence attractiveness for oviposition, especially for specialist insects that are well adapted to their host plants. In this study, we investigated the oviposition preference of *Pieris* butterflies (Lepidoptera: Pieridae) by offering them the choice between 350 different naturally occurring Arabidopsis accessions. Using a genome-wide association study of the oviposition data and subsequent fine mapping with full genome sequences of 164 accessions, we identified *WRKY42* and *AOC1* as candidate genes that are associated with the oviposition preference observed for *Pieris* butterflies. Host plant choice assays with Arabidopsis genotypes impaired in *WRKY42* or *AOC1* function confirmed a clear role for WRKY42 in oviposition preference of female *Pieris* butterflies, while for AOC1 the effect was mild. In contrast, WRKY42-impaired plants, which were preferred for oviposition by butterflies, negatively impacted offspring performance. These findings exemplify that plant genotype can have opposite effects on oviposition preference and caterpillar performance. This knowledge can be used for breeding trap crops or crops that are unattractive for oviposition by pest insects.

## Teaser

*Pieris*butterfly preference for oviposition on naturally occurring and genetically diverse Arabidopsis accessions is associated with *WRKY42*impairment and negatively impacts caterpillar performance.

## Introduction

Insect herbivores consume considerable amounts of plant biomass, causing major crop losses worldwide. To counteract these herbivores, plants have evolved structural and chemical barriers (Pieterse and Dicke, 2007; Verhage *et al.*, 2011). Structural barriers (e.g. trichomes) can contain defensive secondary metabolites, such as toxic glycoside breakdown products, which are released upon tissue damage to hinder the attacking insect (Kliebenstein *et al.*, 2001; Reymond *et al.*, 2004; Frerigmann *et al.*, 2012). When these constitutive structural and chemical barriers are ineffective, a second line of inducible defense can be activated. Plant recognition of insect herbivory triggers the production of phytohormones such as jasmonic acid (JA), salicylic acid (SA), ethylene and abscisic acid, which subsequently leads to the induction and production of defensive compounds to ward off the invading insect (De Vos *et al.*, 2006b; Howe and Jander, 2008; Vos *et al.*, 2015; Erb and Reymond, 2019). Besides the aforementioned glycoside breakdown products, these induced defenses can include the production of other insecticidal toxins, feeding deterrents, and proteinase inhibitors that impair the activity of digestive proteases in the insect gut (Howe and Jander, 2008). Insect herbivore-induced defense signaling extends systemically to undamaged plant parts, thereby protecting the plant against future herbivore damage (De Vos *et al.*, 2006b; Bodenhausen and Reymond, 2007; Howe and Jander, 2008; Soler *et al.*, 2013; Vos *et al.*, 2013). However, over 75% of herbivorous insect species are specialized on their host plants and are well adapted to their structural and chemical defenses, making it difficult to manage pest insect outbreaks (Schoonhoven *et al.*, 2005).

Amongst the most destructive pests on cruciferous plants are caterpillars from the small and large cabbage white butterfly, *Pieris rapae* and *Pieris brassicae* (Lepidoptera: Pieridae; Hopkins *et al.*, 2009; Okamura *et al.*, 2019). These herbivores belong to the order Lepidoptera and are well adapted to their host’s chemical defenses, via either inactivation or evasion of harmful compounds. In the host plant family, Brassicaceae, glucosinolates (i.e. glycosides) play an important role in the chemical defense against pests (Okamura *et al.*, 2019). *Pieris* caterpillars are adapted to glucosinolates by producing a gut enzyme that diverts formation of the toxic isothiocyanate hydrolytic breakdown products to less toxic nitriles (Wittstock *et al.*, 2004; Okamura *et al.*, 2019). In addition, *Pieris* butterflies use glucosinolates as specific feeding and oviposition stimulants through gustatory recognition (Van Loon *et al.*, 1992; Städler *et al.*, 1995; De Vos *et al.*, 2008; Mumm *et al.*, 2008; Hopkins *et al.*, 2009; Ali and Agrawal, 2012).

Before being in direct contact with a host plant, *Pieris* butterflies use visual and volatile cues to detect a potential host plant during flight (Hern *et al.*, 1996; Smallegange *et al.*, 2006; Zheng *et al.*, 2010). It has been suggested that *Pieris* relies on plant color to induce landings, as plant color seems connected to the plant’s nutritional status, which may be an important prerequisite for oviposition (Myers, 1985; Renwick and Radke, 1988; Hwang *et al.*, 2008). Furthermore, *Pieris* is able to learn which optical traits correspond to suitable host plants by contact-chemosensory detection of glucosinolates (Traynier and Truscott, 1991; Bukovinszky *et al.*, 2005). Insights into preventing insect host selection may therefore be one of the cornerstones for the development of sustainable crop protection. This concept is known as antixenosis or non-preference plant resistance.

After egg deposition by *Pieris* butterflies, Arabidopsis plants were shown to recognize egg-derived elicitors, resulting in the subsequent induction of local SA-dependent defenses (Little *et al.*, 2007; Bruessow *et al.*, 2010; Verhage *et al.*, 2010; Fatouros *et al.*, 2012; Lortzing *et al.*, 2019; Stahl *et al.*, 2020). SA antagonizes JA-dependent anti-herbivore defenses, and hence this egg-mediated induction of SA–JA crosstalk gives the newly born caterpillars a head start by suppressing the JA-dependent defenses that are activated when the larvae start to feed (Bruessow *et al.*, 2010). In black mustard (*Brassica nigra*), insect eggs were shown to activate a rapid local cell death (i.e. hypersensitive response) underneath the deposited eggs, resulting in effective removal of the insect eggs (Shapiro and De Vay, 1987; Fatouros *et al.*, 2016). Hence, both *Pieris* and its host plants have evolved several mechanisms to counteract each other.

Plant responses to *Pieris* caterpillar feeding lead to the production of lipoxygenases that are involved in the biosynthesis of JA and other oxylipins, which activate downstream defenses and can also be directly toxic to herbivores (Kessler *et al.*, 2004; Howe and Jander, 2008; Dabrowska *et al.*, 2009; Shabab *et al.*, 2014). To counteract these plant defenses, compounds in the oral secretions of *Pieris* caterpillars can modulate the plant’s hormone-regulated defense response to the advantage of the insect (Verhage *et al.*, 2011; Broekgaarden *et al.*, 2015).

More recently, it was shown that the preference and performance hypothesis, which states that female butterflies prefer to oviposit on host plants that are best for their offspring, could partially be explained by plant responses to oviposition (Griese *et al.*, 2020). Plants that were preferred for oviposition resulted in better caterpillar performance (i.e. weight gain). In an oviposition assay with predominantly non-domesticated ­Brassicaceae plant species, *P. rapae* butterflies clearly preferred to oviposit on black mustard (*Brassica nigra* L.) plants, which develop a hypersensitive response to insect eggs, leading to necrosis of plant material underneath deposited eggs. Although egg survival was lower, *P. rapae* caterpillars gained significantly more weight on plants expressing an egg-induced hypersensitive response, demonstrating a positive correlation between oviposition preference, egg-induced hypersensitive response, and caterpillar performance.

Genome wide association studies (GWAS) have extensively been used to gain insight into naturally evolved plant adaptive responses, revealing genes with important functions in diverse processes of plant growth and survival (Atwell *et al.*, 2010; Baxter *et al.*, 2010; Kloth *et al.*, 2016; Davila Olivas *et al.*, 2017b; Thoen *et al.*, 2017; Proietti *et al.*, 2018; Coolen *et al.*, 2019). With the ultimate aim to discover novel plant traits that make a plant (un)favorable for host selection by specialist herbivores such as *Pieris*, we mined the natural genetic variation amongst 350 naturally occurring Arabidopsis accessions for genomic regions that are related to oviposition discrimination by *P. rapae.* To this end, we offered *P. rapae* butterflies a choice out of 350 randomly placed Arabidopsis accessions and scored the number of eggs laid per accession. The obtained data were subsequently used in a GWAS followed by preference and performance validation experiments with mutants of selected candidate genes.

## Materials and methods

### Plant material

In this study 350 Arabidopsis accessions of the haplotype map (HapMap) population (http://bergelson.uchicago.edu/wp-content/uploads/2015/04/Justins-360-lines.xls) were used. The HapMap population has been genotyped for 250 000 bi-allelic single nucleotide polymorphism (SNPs; Baxter *et al.*, 2010; Platt *et al.*, 2010; Chao *et al.*, 2012) and after quality control and imputation this SNP set was reduced to a set of 214 051 SNPs (Thoen *et al.*, 2017). An Arabidopsis T-DNA insertion line in the Col-0 background for *WRKY42* (SALK_121674C; designated ‘*wrky42*’) was selected according to fine mapping and subsequent amino acid change results (see Figs 2, 3). The insertion line was obtained from the Nottingham Arabidopsis Stock Centre (NASC) and subsequently genotyped to obtain a homozygous line. MYC-triple mutant *myc234*, AOS mutant *aos* (*dde2-2*), and *AOC*::RNAi (line *16-1*; Leon-Reyes *et al.*, 2010) were kindly provided by Roberto Solano, Beat Keller, and Claus Wasternack (Von Malek *et al.*, 2002; Delker, 2005; Fernandez-Calvo *et al.*, 2011).

**Fig. 1.**
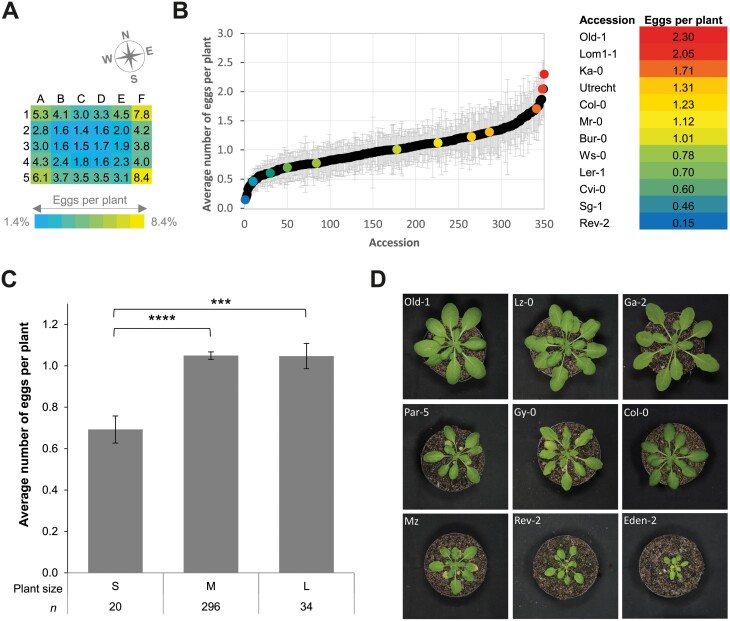
Oviposition distribution, natural variation, and the relationship between plant size in oviposition preference by *P. rapae* butterflies on Arabidopsis accessions. (A) Heatmap showing the average number of eggs deposited per plant in the respective plots over seven independent experiments. The position of the cage is indicated by the compass. (B) Normalized average number of eggs deposited by *P. rapae* butterflies on 350 different Arabidopsis accessions. Data are the average of seven independent experiments on the total set of 350 accessions, each experiment containing one randomly positioned plant per accession. In each experiment, 10–15 female *P. rapae* butterflies were allowed to freely oviposit for 2–3 d on the offered population of 350 plants. Error bars show standard errors (±SE). In the color gradient on the right, specific accessions with distinct normalized average egg counts are highlighted. (C) Normalized average number of eggs deposited per plant category (*n*=4) on small (S), medium (M), and large (L) plants with standard error (±SE) bars. Significance was calculated using Student’s *t*-test (***P≤0.001 and ****P≤0.0001). (D) Examples of 4-week-old Arabidopsis accessions in the plant size categories.

**Fig. 2.**
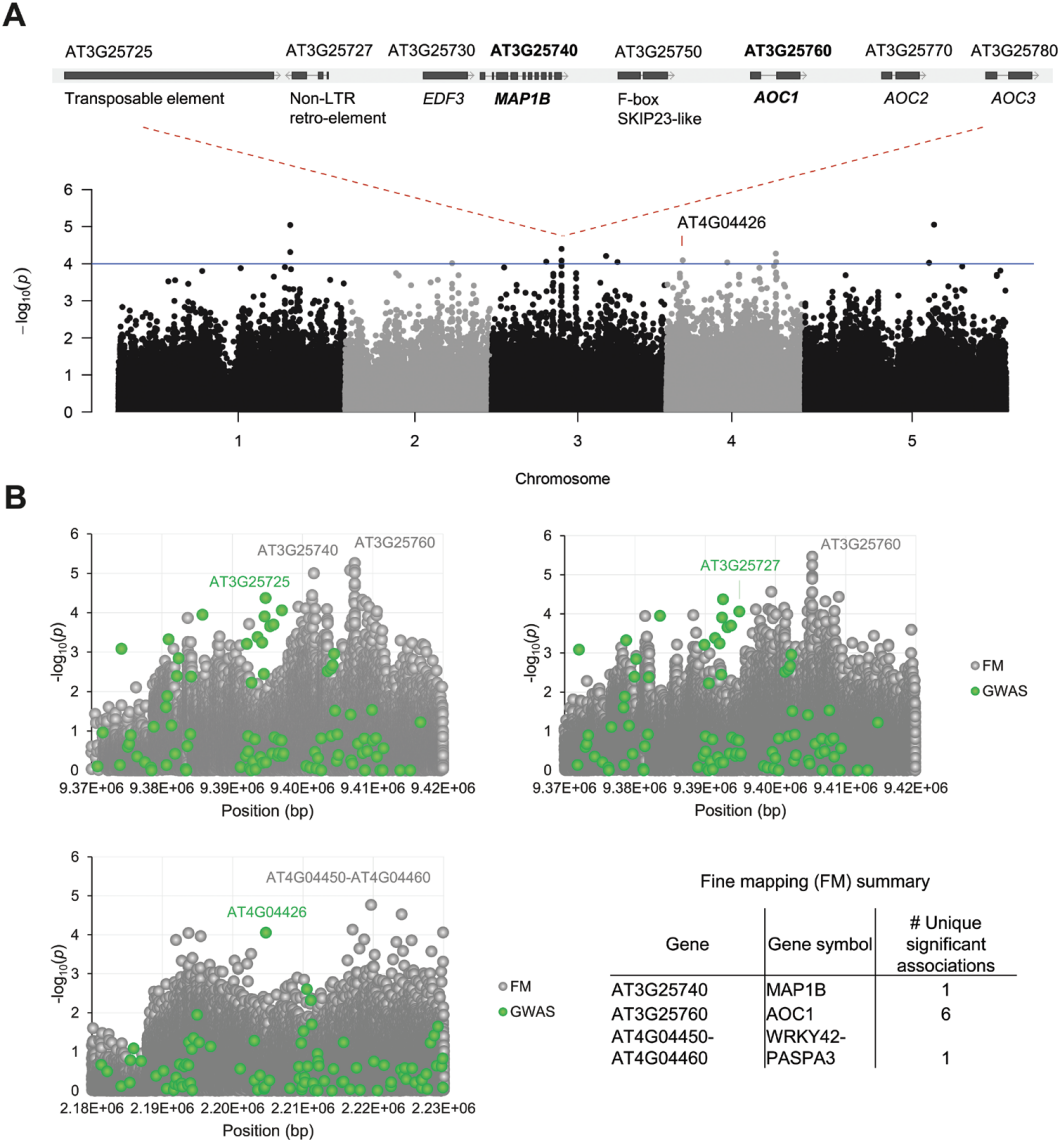
GWAS and fine mapping results for oviposition preference of *P. rapae* on 346 Arabidopsis accessions. (A) Manhattan plot (grey and dark grey) showing the –log_10_(*p*) values of the SNP–trait associations from the GWAS results on Arabidopsis chromosomes 1–5 (*x*-axis). Narrow sense heritability was estimated to be very low (*h*^*2*^=0.0014 with a 95% confidence interval of 0.0–1.0; Kruijer *et al.*, 2015). The blue line indicates the arbitrary LOD threshold of 4.0 (–log_10_(*p*)=4.0) for selection of SNPs. Above the Manhattan plot a gene cluster is depicted (http://signal.salk.edu/atg1001/3.0/gebrowser.php) that was found upstream of the transposable element gene *AT3G25725* in which SNPs above the threshold were found by the GWAS. In bold three GWAS loci are indicated for which SNP–trait associations (LOD≥4.0) were confirmed via fine mapping. LTR, long terminal repeat. (B) Fine mapping (FM; grey dots) of three SNP–trait associations that were identified by the GWAS (green dots), using the 50-kb window around the GWAS SNPs from the genome sequences of 164 of the tested Arabidopsis accessions. The graphs show the –log_10_(*p*) values of the SNP–trait associations on the *y*-axis and the chromosome position of the SNPs in base pairs (bp) on the *x*-axis. Significant (FDR-corrected) FM associations are shown in black ATG numbers along with the number of significant associations in the FM summary.

**Fig. 3.**
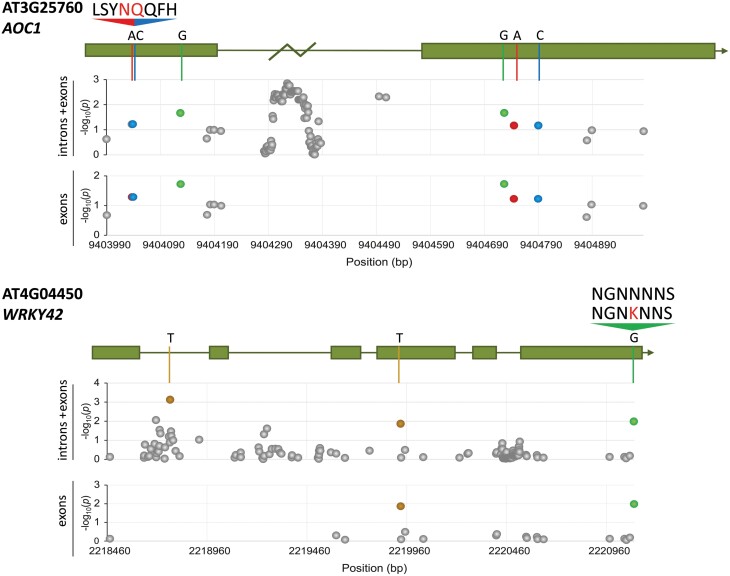
Amino-acid changes confirm possible altered gene function of fine mapping candidates. Manhattan plots of fine mapping results (MAF>5%, FDR-corrected) for the candidate genes *AT3G25760* and *AT4G04450*, including both introns and exons or only exons. *y*-Axes show the –log_10_(*p*) and the *x*-axes the position in base pairs (bp). For each gene a model of the introns and exons is shown according to the 1001 genomes browser (http://signal.salk.edu/atg1001/3.0/gebrowser.php). A zigzag indicates missing sequencing data (*AT3G25760*). Important (significant) nucleotides are indicated with colors: A (red), T (ochre/yellow), C (blue), and G (green). Non-synonymous amino acid changes are depicted with red letters.

### DNA isolation and T-DNA genotyping

Plant DNA was obtained by grinding (~5 mg) frozen leaf material using a Qiagen TissueLyser and a Sucrose Prep method (Berendzen *et al.*, 2005). Homozygous T-DNA insertion mutant plants were identified and genotyped by PCR, using Phire Hot Start II DNA Polymerase (Thermo Fisher Scientific), according to manufacturer’s instructions. The T-DNA left border primer LBb1.3 (ATTTTGCCGATTTCGGAAC) was used in combination with a right border primer for *WRKY42* (TTTGTGCGTCTGTTACGTACG) to genotype homozygous T-DNA plants. Wild type plants were genotyped with the latter right border primer and left border primer of *WRKY42* (TGCAACGGTAATAAGCTCGAG).

### Plant growth conditions

Arabidopsis seeds were sown in cultivation containers filled with autoclaved river sand supplied with half-strength Hoagland solution containing chelated iron (i.e. sequestrene) as described by Van Wees *et al.* (2013), to prevent iron deficiency and subsequent chlorosis (i.e. yellowing). Cultivation containers were enclosed in a tray with water and covered with a transparent lid to attain a high relative humidity (RH) for germination. Seed stratification was performed in the dark for 2 d at 4 °C to ensure a homogeneous germination. After stratification, the trays were moved to a growth chamber with an 8 h day–16 h night cycle, a temperature of 21 °C, and a light intensity of 100 µmol m^−2^ s^−1^. Tray lids were slightly opened after 8 d and gradually removed over a 2-day period to adjust to the 70% RH present in the growth chamber, which is commonly used for Arabidopsis. Two-week-old seedlings were transplanted to individual pots containing an autoclaved mixture of river sand and potting soil (1:1 (v:v)). Plants were supplied with water from the bottom up three times per week, and at an age of 3 weeks the plants were supplied once with half-strength Hoagland solution (10 ml/plant).

### Rearing of *Pieris*

*Pieris rapae* was reared on cabbage plants (*Brassica oleracea* convar. capitata var. alba) under greenhouse conditions (24 °C, with natural daylight). Butterflies were supplied with flowering plants such as *Lantana camara* for their (nectar) food and additionally with a solution of 20% honey and 10% sucrose. Inbreeding of the population was minimized by regularly adding wild butterflies and caterpillars collected in the Dutch Flevopolder to the existing population. *Pieris brassicae* was reared in a climate-controlled room at 22 ± 2 °C, a light–dark regime of 16:8 h, and 50–70% relative humidity on Brussels sprout plants (*B. oleracea* var. gemmifera cv Cyrus) as described by Karssemeijer *et al.* (2022).

### Oviposition preference tests (350 accessions)

Oviposition by *P. rapae* was performed in seven independent experiments, each with a single 4-week-old plant of all 350 Arabidopsis accessions. The 350 plants were randomized and assigned to 1 of the 30 square 27.5 × 27.5 cm plots (A1–F5) in a 2.0 × 1.6 m (1.2 m high) insect cage in the greenhouse (Supplementary Fig. S1). Accessions were evenly spaced in the cage. A mixed group of approximately 20–30 male and female butterflies was released into the cage and females were allowed to oviposit freely on the 350 accessions for 2–3 d, depending on the weather conditions. Butterfly feeding sites consisted of a solution containing 20% (v/v) honey and 10% (w/v) sucrose, which were positioned in the middle of cage locations B2, E2, B4, and E4. After 2–3 d the butterflies were removed from the cage after which the number of eggs was recorded by counting all the eggs on both the plant and the corresponding pot (Supplementary Tables S1, S2).

### Oviposition preference tests (mutants and small subsets of accessions)

For the experiments in which the preference of *P. rapae* or *P. brassicae* butterflies was tested on subsets of Arabidopsis accessions or mutants, small 30 × 30 cm (54 cm high) insect cages (*n*=5–22 cages) were used with one to two female butterflies and feeding solution in the middle. Egg numbers were recorded when eggs were deposited on the plant or the corresponding pot after a maximum of 2–3 d. For the comparison between Col-0 (with trichomes) and Col-5 (glabrous), each test contained two Col-0 plants and two Col-5 plants. Mutant tests contained one control plant and one mutant plant per cage. For testing the oviposition preference of *P. brassicae* on a subset of 11 accessions of the HapMap collection, a similar approach was used. Plants were randomized throughout the small cage and egg numbers were recorded after 2–3 d. Egg numbers were not corrected for position effects in these small cage tests since no clear location effects were observed and plants were on opposite or randomized positions in each test.

### Caterpillar performance test

Freshly hatched *P. brassicae* neonate caterpillars were gently picked up with a brush and placed on either one 5-week-old Arabidopsis wild-type Col-0 plant or a mutant *wrky42* plant, from which *P. brassicae* eggs were removed 24–48 h after deposition and prior to caterpillar placement. Each plant with one caterpillar was contained in a plastic cup covered with mesh to prevent caterpillars from escaping. Plants were replaced with fresh new plants well before caterpillars would finish the plant, in order to prevent starvation effects. After 13 d caterpillars were weighed.

### Plant size categories

Plant sizes were evaluated for four out of the total of seven experiments. Plants were categorized in three size classes that correspond to plant rosette diameter respective to the pot size (Ø=5.5 cm): category 1 (rosette fully within the pot boundary, <5.5 cm), category 2 (max. four rosette leaves exceeding the pot boundary, ~5.5 cm), and category 3 (more than four rosette leaves exceeding the pot boundary, >5.5 cm). To combine the average plant size parameters over the four experiments, the three categories were assigned the numbers 1, 2, and 3, respectively, after which the average plant size category per accession was calculated. For the correlation analysis with egg counts, accessions with an average plant size ≤1.5 were placed in bin ‘small’, accessions with average plant size >1.5 and ≤2.5 were placed in bin ‘medium’, and accessions with average plant size >2.5 were placed in bin ‘large’ (Supplementary Table S2).

### Genome-wide association study

GWAS was performed using data of 346 accessions on the average number of deposited eggs per plant that was normalized for the cage position effects (raw egg number/average number of eggs per plot position (A1–F5; Supplementary Fig. S1), i.e. ‘position correction factor’, resulting in ‘cage position corrected data’) and the total number of eggs deposited per experiment (‘cage position corrected data’ × ‘correction factor’ of total number of eggs per experiment, resulting in ‘normalized data’), and subsequently transformed to a normal distribution, using an arcsine transformation (Supplementary Tables S2, S3). The transformed phenotype was defined as arcsin(√(average normalized number of eggs per plant/6)). GWAS was employed using Fast-LMM software (Lippert *et al.*, 2011) with a minor allele frequency (MAF) of >0.05 together with an arbitrary threshold with a logarithm (base 10) of the odds (logarithm of odds (LOD); −log_10_(*p*)) score of 4 to determine SNP associations of interest. Linkage disequilibrium was taken into account by including all SNPs within 25 kb up- and downstream of the SNP of interest. Narrow sense heritability was estimated using the ‘heritability’ R package (Kruijer *et al.*, 2015).

### Fine mapping

Fine mapping was performed using full 50-kb genome sequences available from the 1001 genomes project (Weigel and Mott, 2009; http://signal.salk.edu/atg1001/3.0/gebrowser.php). Genome sequences were formatted into a nucleotide matrix for all 164 accessions using Jalview (http://www.jalview.org/; Waterhouse *et al.*, 2009). Locus specific mapping was performed using a MAF of >0.05. A Kruskal–Wallis test was used for obtaining significant, false discovery rate (FDR)-corrected, SNP–trait associations using R and the ‘p. adjust’ function with the Benjamini–Hochberg method (Benjamini and Hochberg, 1995). For fine mapping of nucleotide changes within genes, nucleotide sequences were first aligned, making sure that no shifts were present due to insertions and deletions.

## Results

### *Pieris rapae* oviposition is influenced by edge effects and natural sunlight

To study the genetic basis of host selection for oviposition by *Pieris* butterflies, we investigated their oviposition preference when offered 350 naturally occurring accessions of the Arabidopsis HapMap collection (Baxter *et al.*, 2010; Platt *et al.*, 2010). *Pieris rapae* butterflies were allowed to oviposit on the total collection of randomly distributed and equally spaced Arabidopsis accessions for 2–3 d, in a cage set-up of seven independent randomized experiments, resulting in a total number of 622–1879 eggs deposited per experiment (Fig. 1A; Supplementary Fig. S1; Supplementary Tables S1, S2). Since *P. rapae* is known to oviposit predominantly on sunny and warm days during the morning and early afternoon and our experimental butterfly cage was positioned in a greenhouse with natural daylight, we anticipated that the position of the plants within the cage could influence the choice of host plant by the butterflies (Root and Kareiva, 1984). Figure 1A shows that plant position influenced host plant choice and in our cage set-up most eggs were deposited on the east side of the cage, i.e. the side where the sunlight was coming from in the morning. Oviposition preference was also influenced by the corners and the edges of the cage, as the majority of the eggs were deposited in these zones of the cage (Fig. 1A). Natural flight and search behavior of *P. rapae* butterflies was previously described to cause ‘edge effects’ under field conditions (Root, 1973; Jones, 1977; Muriel and Grez, 2002), which could explain the observed skewed egg distribution over the cage.

### *Pieris rapae* oviposition is influenced by morphology and other natural genetic variation in Arabidopsis accessions

To analyse the effect of plant genotype on the oviposition preference of *P. rapae* butterflies, we first normalized the egg counts per accession for the average cage-position effects in each experiment and subsequently normalized that data for the total egg counts per experiment (Supplementary Table S2). The resulting normalized average egg counts per accession are depicted in Fig. 1B. To test if the size trait influenced *P. rapae* oviposition, we categorized the accessions of four experiments into three size classes that correspond to plant rosette diameter with respect to the pot size (Ø=5.5 cm): category 1 (<5.5 cm), category 2 (~5.5 cm), and category 3 (>5.5 cm). The average plant size within each category positively correlated with the normalized average number of eggs per corresponding classes (Pearson correlation; *R*=0.90), with medium and large plants receiving significantly more eggs than small plants (Fig. 1C). The Rev-2 accession received a minimal number of eggs, whereas Old-1 received the most eggs. Morphologically these plants are very different, possibly explaining the difference in *P. rapae* preference. Under the growth conditions used, accession Old-1 is a large plant, whereas accession Rev-2 is a small plant (Fig. 1D).

Amongst the 350 accessions, we also observed two glabrous (i.e. trichome-less) accessions, Est-0 and Br-0 (Hauser *et al.*, 2001; Barth *et al.*, 2002; Bloomer *et al.*, 2012). Previously, Reymond *et al.* (2004) demonstrated that *P. rapae* caterpillars performed better on glabrous plants, and hence we hypothesized that butterflies may anticipate this and prefer to oviposit on plants without trichomes. With a normalized egg score of 1.05 and 1.21, respectively, Br-0 and Est-0 indeed belong to the accessions that received above median numbers of eggs per plant of tested Arabidopsis accessions. However, additional experiments with trichome-containing Col-0 and its natural glabrous mutant Col-5 showed that *P. rapae* butterflies did not prefer to oviposit on glabrous plants over plants with trichomes in our experimental set-up (Supplementary Fig. S2).

Fourteen accessions developed spontaneous chlorosis (i.e. yellowing) and necrosis under our growth conditions, which was previously described by Todesco *et al.* (2010) as being late-onset necrosis. Of these accessions, none with moderate or severe late-onset necrosis was found amongst the top 25% of most-preferred accessions for oviposition, whereas 6 of the 14 were amongst the top 25% of least-preferred accessions (Supplementary Table S2). This suggests that spontaneous necrosis of the plant is an unfavorable trait for host selection by *P. rapae* butterflies.

Previously, Kliebenstein *et al.* (2001) measured aliphatic- and indole-glucosinolate levels in a range of Arabidopsis accessions, 15 of which were also present amongst the 350 accessions tested in this study (Supplementary Table S2). The class of indole-glucosinolates has been associated with enhanced oviposition preference by *P. rapae* (Huang and Renwick, 1994; De Vos *et al.*, 2008; Müller *et al.*, 2010). Comparing the normalized average number of eggs deposited on these 15 accessions with the aliphatic- and the indole-glucosinolate levels reported by Kliebenstein *et al.* (2001) revealed a moderate non-significant positive correlation (*R*=0.45) between egg number and indole-glucosinolate levels, which points in the same direction as previous findings. Conversely, we found a significant (*P*<0.05) moderate to strong negative correlation (*R*=−0.61) between egg number and aliphatic-glucosinolate levels, which suggests that this class of glucosinolates are negative oviposition cues for *P. rapae* butterflies.

### Genome-wide association study reveals Arabidopsis loci associated with *P. rapae* oviposition

To unravel the underlying host plant genetics that influences *P. rapae* host plant choice, we mined the natural genetic variation in egg deposition among the tested Arabidopsis accessions for genetic components that contribute to the observed oviposition differences. We performed a GWAS on the normalized and transformed data of seven independent experiments (Supplementary Tables S2, S3). We performed a GWAS using the factored spectrally transformed linear mixed models (FaST-LMM) algorithm (Lippert *et al.*, 2011) and a set of ~214 000 SNPs (Baxter *et al.*, 2010; Platt *et al.*, 2010; Chao *et al.*, 2012; Thoen *et al.*, 2017). SNP–trait associations of interest were selected by setting an arbitrary threshold with a LOD (−log_10_(*p*)) score of 4.0. GWAS results revealed 11 SNP–trait associations for a total of 10 unique loci (Fig. 2A; Table 1) and 56 SNPs in linkage disequilibrium (estimated to be 10–50 kb; Nordborg *et al.*, 2005; Kim *et al.*, 2007) accounting for an additional 25 loci (Supplementary Table S4). Some of the genes within these loci (LOD ≥4) have previously been connected with plant traits affecting herbivory, stress signaling, and general defensive mechanisms (Supplementary Table S4).

**Table 1.** GWAS candidate loci associated with *P. rapae* oviposition preference

**Gene**	**LOD score**	**TAIR gene description**	**SNPs**
*AT1G62370*	4.31/5.04	RING/U-box superfamily protein	2
*AT3G20990*	4.02	Copia-like retrotransposon family	1
*AT3G25725*	4.37	Copia-like retrotransposon family	1
*AT3G25727*	4.06	RNA-directed DNA polymerase (reverse transcriptase)	1
*AT3G43460*	4.18	Unknown protein	1
*AT3G46010*	4.02	Actin-depolymerizing factor 1 (ADF1)	1
*AT4G04426*	4.06	Copia-like retrotransposon family	1
*AT4G30080*	4.31	Auxin response factor 16 (ARF16)	1
*AT4G30110*	4.00	Arabidopsis heavy metal ATPase 2 (HMA2)	1
*AT5G43130*	5.05	TBP-associated factor 4 (TAF4)	1

### Fine mapping reveals candidate genes associated with *P. rapae* oviposition preference

To tune in on the candidate genes located in the oviposition preference-associated loci, additional fine mapping was performed using full genome sequences of 164 accessions (Supplementary Table S5) available via the 1001 genomes project (Weigel and Mott, 2009). The fine mapping procedure makes use of all genetic variances within the selected genomic regions of the 164 full genome sequences, while the GWAS was based on the SNPs within the accessions relative to the reference genome of accession Col-0. Because linkage disequilibrium in Arabidopsis is estimated to be 10–50 kb (Nordborg *et al.*, 2005; Kim *et al.*, 2007), a 50–kb window surrounding the SNPs of interest was included for finding genes of interest. Fine mapping was based on a Kruskal–Wallis test for trait associations with a minor allele frequency larger than 5% (MAF>0.05) (Zhao *et al.*, 2007). Fine mapping results show that there are several significant false discovery rate (FDR)-corrected associations that correspond to the loci identified with our GWAS (Fig. 2B; Supplementary Fig. S3). On the locus surrounding transposable element gene *AT3G25725* on chromosome 3, associations were found in an upstream cluster containing four genes involved in ethylene signaling and JA biosynthesis (Fig. 2A). Just upstream of *ETHYLENE RESPONSE DNA BINDING FACTOR 3* (*EDF3*, *AT3G25730*) and downstream of JA biosynthesis gene *ALLENE OXIDE CYCLASE 2* (*AOC2*, *AT3G25770*) and *AOC3* (*AT3G25780*), two significant SNP peaks were observed with fine mapping (Fig. 2B), pointing to *AOC1* (*AT3G25760*) and *METHIONINE AMINOPEPTIDASE 1B* (*MAP1B*, *AT3G25740*). Of these, *AOC1* is one of the four genes in Arabidopsis that encodes an allene oxide cyclase, which catalyses an essential step in JA biosynthesis (Wasternack and Hause, 2013; Zhang *et al.*, 2020). Downstream of *AOC1*, a significant association was found with *MAP1B*, encoding a methionine aminopeptidase that was shown to be a potential target of miRn5998, a microRNA responsive to JA treatment (Zhang *et al.*, 2012). On chromosome 4 a significant association was observed at the interval of transcription factor gene *WRKY42* (*AT4G04450*) and *PUTATIVE ASPARTIC PROTEINASE A3* (*PASPA3*, *AT4G04460*). *WRKY42* encodes a WRKY transcription factor that was shown to be involved in plant phosphate (P_i_) homeostasis and modulation of SA and reactive oxygen species in leaf senescence (Su *et al.*, 2015; Niu *et al.*, 2020).

### Amino acid changes support fine mapping results for *AOC1* and *WRKY42*

To further substantiate the candidate genes that were identified through fine mapping (Fig. 2), we analysed *AOC1* and *MAP1B* on chromosome 3, and *WRKY42* and *PASPA3* on chromosome 4 for alterations in nucleotide sequences that lead to amino acid changes in the translated protein with potential impact on protein function, using the 164 full Arabidopsis genomes (Fig. 3; Supplementary Fig. S4). *MAP1B* on chromosome 3 and *PASPA3* on chromosome 4 did not contain SNPs that result in non-synonymous amino acid changes. However, *AOC1* on chromosome 3 and *WRKY42* on chromosome 4 displayed natural genetic variation with potential impact on gene regulation or protein function. For *AOC1*, comparison of the 164 genomes resulted in 58 significant associations within the intron region that might alter *AOC1* expression within the different Arabidopsis accessions. Furthermore, within the exons we found six significant associations of which two result in non-synonymous amino acid changes (LSY*SK*QFH→LSY*NQ*QFH) in the first exon and thus can potentially alter protein structure and functioning. For *WRKY42*, comparison of the 164 genomes resulted in one significant association in the first intron and two significant associations in the exons of which one is a non-synonymous amino acid change in the last exon (NGN*N*NNS→NGN*K*NNS) that can potentially alter protein function. Hence, *AOC1* and *WRKY42* were selected for further validation of their role in oviposition preference.

### *AOC* and *WRKY42* are involved in oviposition preference by *Pieris* butterflies

To validate host plant choice by *Pieris* butterflies for the selected genes, an available *AOC::RNAi* line (line *16-1*; Delker, 2005) with diminished AOC protein synthesis (including AOC1 and partially redundant AOC2, 3, and 4; Leon-Reyes *et al.*, 2010; Stenzel *et al.*, 2012) and a *WRKY42* T-DNA insertion line (allele knockout, *wrky42-1*; Niu *et al.*, 2020) were used in oviposition assays together with wild-type Col-0 plants. A MYC triple-mutant (*myc234*; Fernandez-Calvo *et al.*, 2011), lacking glucosinolates and affected in JA responsiveness, was taken along as negative control since selection of a suitable brassicaceous host plants by *Pieris* butterflies occurs via gustatory sensing of glucosinolates (De Vos *et al.*, 2008; Mumm *et al.*, 2008; Hopkins *et al.*, 2009; Ali and Agrawal, 2012; Schweizer *et al.*, 2013). As a control for the effect of JA-dependent plant defenses upstream of *AOC1* in the JA-biosynthesis pathway, *ALLENE OXIDASE SYNTHASE* (*AOS*) mutant plants (i.e. *aos or* delayed-dehiscence2-2*; dde2-2*) lacking JA were taken along (Von Malek *et al.*, 2002; Koo, 2017).

Due to persistent rearing problems with *P. rapae* caused by a viral infection in the rearing population, butterflies of closely related *P. brassicae* were used for the validation experiments. Beforehand, oviposition preference of *P. brassicae* was assessed on a subset of 11 accessions from the HapMap collection for comparison with *P. rapae* oviposition preference (Supplementary Fig. S5). Although *P. brassicae* oviposits in clusters of eggs in contrast to *P. rapae*, which oviposits with single eggs, oviposition on the subset of the 11 selected accessions showed a similar trend with the Rev-2 accession receiving the lowest number of eggs and the Old-1 accession receiving the second-highest number of eggs (Supplementary Fig. S5).

**Fig. 4.**
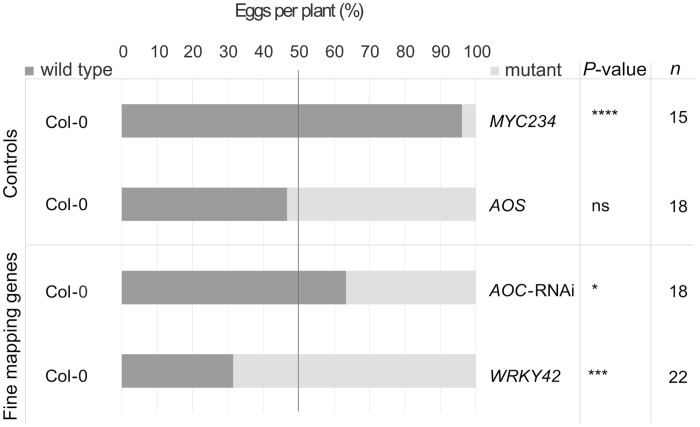
Oviposition choice assay on fine-mapping confirmed genes. Six mutants, including a *MYC*-triple mutant (*myc234*) that lacks glucosinolates and a JA lacking mutant (*aos*) control, a T-DNA insertion line (*wrky42*) and an RNAi line (*AOC*::RNAi line 16-1) were tested for oviposition preference by *P. brassicae* in a two-plant choice assay. Replicates of each two-plant assay are indicated as *n* (*n*=15–22). Bars represent the average distribution of eggs between wild-type Col-0 plants (dark grey bars) and the mutant plants (light grey bars) in a choice test. Significant differences were calculated using Student’s *t*-test (non-significant, ns; *P≤0.05, **P≤0.01, ***P≤0.001, and ****P≤0.0001).

Mutant *myc234* plants were highly unattractive for oviposition, likely due to the lack of glucosinolates that *Pieris* butterflies use to select suitable host plants (Fig. 4). Surprisingly, the JA biosynthesis mutant *aos* showed equal attractiveness for oviposition to wild-type Col-0 plants. This may be explained by the fact that unlike *myc234* plants, *aos* plants have similar basal glucosinolate levels and volatile emissions as Col-0 (Snoeren *et al.*, 2009; Pangesti *et al.*, 2016).

In the oxylipin biosynthesis pathway, AOS acts directly upstream of AOC and their combined action results in the biosynthesis of *cis*(+)-12-oxo-phytodienoic acid (OPDA), the precursor of JA (Howe and Schilmiller, 2002). Hence, one would expect that the *AOC*::RNAi line, which has reduced levels of AOC protein (including AOC1), would behave similarly to *aos* in terms of effects on oviposition preference. However, in contrast to *aos*, the *AOC::*RNAi line was slightly less attractive to butterflies for oviposition than wild-type Col-0 plants. In the JA biosynthesis pathway, AOS converts 13-hydroperoxylinolenic acid into the unstable intermediate 12,13-epoxy octadecatrienoic acid (12,13-EOT), which is then converted by AOC into *cis*(+)-12-OPDA. Chemical *in vitro* experiments, in the absence of AOC, showed that 12,13-EOT non-enzymatically transforms to (i) α- and γ-ketols through hydrolysis and (ii) racemic 12-OPDA through cyclization (Brash *et al.*, 1988; Song and Brash, 1991). The physiological significance of α- and γ-ketols and racemic 12-OPDA is unclear, but both products or their downstream stimulated secondary metabolites are expected to accumulate in the *AOC*::RNAi line used in our study, and can possibly explain the difference between *aos* and *AOC::* RNAi in terms of oviposition preference. However, the effect of oviposition preference in the *AOC::*RNAi-Col-0 choice assay was rather mild. Hence, future research with additional *AOC* perturbed genotypes should shed more light on this matter.

Mutant *wrky42* plants received significantly more eggs than wild-type plants (Fig. 4), confirming the involvement of *WRKY42* in host preference by *Pieris* butterflies. This observation might be related to the observation by Niu *et al.* (2020) that *WRKY42* overexpression promotes age-dependent leaf senescence that is accompanied by leaf yellowing, which is an unattractive plant trade for butterflies as we have suggested before (Supplementary Table S2). Furthermore, mutant *wrky42* plants were also shown to have an increased chlorophyll content (Niu *et al.*, 2020), which might be attractive to butterflies. Niu *et al.* (2020) also showed that the SA content was significantly lower in mutant *wrky42* plants, which could potentially alter the volatile composition including methyl salicylate (MeSA). Groux *et al.* (2014) demonstrated that MeSA acts as oviposition repellent for *P. brassicae* butterflies, possibly explaining the significant increase in oviposition attractiveness of mutant *wrky42* plants.

### Caterpillar performance is affected in mutant *wrky42* plants

**Fig. 5.**
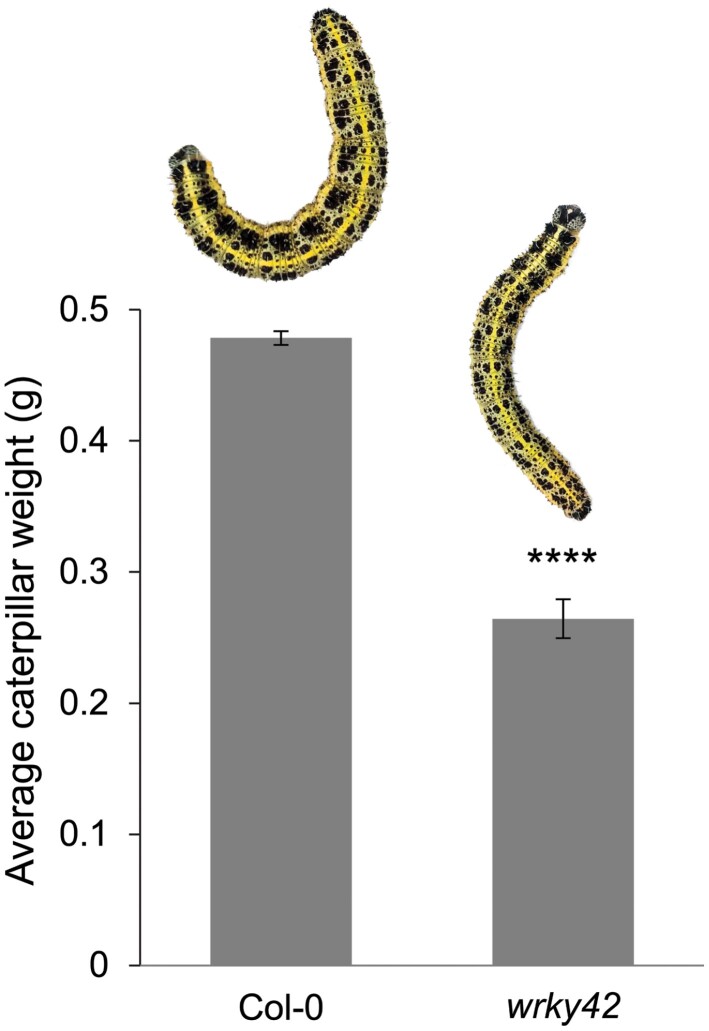
Caterpillar performance on mutant *wrky42*. Graph showing the average caterpillar weight in grams along with standard error (±SE) of mean error bars in a no-choice test. Caterpillar weight was measured after placing one L1 *P. brassicae* caterpillar on either a mature wild-type Col-0 plant (*n*=13) or a *wrky42* mutant plant (*n*=14), from which deposited eggs were removed prior to caterpillar exposure, and allowing them to feed for 13 d. Plant replacements were added well before food was becoming scarce. Significant differences were calculated using Student’s *t*-test (****P≤0.0001).

According to the mother knows best (i.e. preference performance) hypothesis, offspring were expected to perform better on mutant *wrky42* plants than on wild-type Col-0 plants. To test this hypothesis, a performance test was conducted with the *wrky42* mutant, since this line gave the highest average preference for oviposition, with highest significance. Caterpillar performance (i.e. growth rate) on plants of which egg depositions were removed previously was significantly decreased by 45% on *wrky42* compared to caterpillar performance on wild-type Col-0 plants (Fig. 5). Thus, the mother knows best hypothesis is not supported by our findings, indicating that in this specific case butterflies preferred to oviposit on plants that do not support their offspring.

## Discussion

### The study system

To study the contribution of plant genes to oviposition preferences by *Pieris* butterflies we studied the natural genetic variation in the model plant Arabidopsis. It has been questioned whether there has been an evolutionary arms race between Arabidopsis and *Pieris* because of their separation in seasonal occurrence (Harvey *et al.*, 2007). However, especially summer annuals within the HapMap collection used in this study might have experienced selective pressure by herbivores such as *P. rapae* (Pigliucci, 1998; Johanson *et al.*, 2000; Koornneef *et al.*, 2004; Edger *et al.*, 2015; Davila Olivas *et al.*, 2017a). Notwithstanding the fact that Arabidopsis–*Pieris* interactions are found infrequently in nature, both species do interact and display responses that are typical for diverse plant–insect interactions (Bodenhausen and Reymond, 2007; Okamura *et al.*, 2019). We therefore set out to study this interaction by mining the natural genetic variation in the Arabidopsis HapMap collection for traits affecting oviposition preference.

### Host selection by *Pieris* butterflies

Host selection is one of the crucial steps in insect–plant interactions in which plant traits can affect both plant and insect herbivore survival. For insect herbivores such as *Pieris*, both visual and non-visually perceived plant traits (i.e. gustatory and contact-chemosensory detection) can influence host selection as was shown by many studies (Traynier and Truscott, 1991; Van Loon *et al.*, 1992; Städler *et al.*, 1995; Hern *et al.*, 1996; Bukovinszky *et al.*, 2005; Smallegange *et al.*, 2006; De Vos *et al.*, 2008; Mumm *et al.*, 2008; Hopkins *et al.*, 2009; Zheng *et al.*, 2010; Ali and Agrawal, 2012). Plants within the HapMap collection of 350 accessions displayed a wide variety of plant shapes and sizes, which can influence host selection by *Pieris* butterflies and may or may not have been coupled to plant defense-related traits. Depending on the weather conditions, being warm and sunny preferably, butterflies were allowed to oviposit on the plants for 2–3 d, potentially allowing for learning behavior (Traynier and Truscott, 1991; Bukovinszky *et al.*, 2005). The relatively long host selection period might also have influenced host selection since already deposited eggs may have triggered plant defenses that could potentially have been communicated to other plants via volatile compounds.

### Cage set-up

Results from the host selection experiments show that butterflies responded strongly to edges and especially corners in our large-scale cage set-up (Fig. 1A; Supplementary Fig. S1; Supplementary Table S1). These effects were shown to be even stronger on the east side of the set-up where natural daylight entered during the mornings when *Pieris* is most actively ovipositing (Root and Kareiva, 1984). Furthermore, the overall number of eggs deposited on the 350 plants differed per experiment, ranging by a factor of 3 among experiments (Supplementary Table S2). The latter is most likely dependent on presence of morning sun on experimental days. These results show how important it is to carefully monitor experimental set-ups and insect behavior, and correct the data for confounding effects, before interpreting the data and using it for further study.

### Plant size matters for *Pieris* oviposition

Based on a number of general observations in the collection of 350 Arabidopsis accessions, we explored the dataset by asking a number of specific questions related to the effect of plant size, the role of trichomes, spontaneous chlorosis, and glucosinolate profiles on the oviposition preference of *Pieris* (Fig. 1C, D; Supplementary Table S2). We found that small plants received significantly fewer eggs than medium and large plants. This can be explained by the fact that larger plants have simply more leaf surface and so by chance would have a higher probability of being chosen by the butterflies. However, it may also be adaptive to oviposit on larger plants, because they would provide the offspring with more food.

### Plant trichomes have no clear role in oviposition by *Pieris* butterflies

We also tested the effect of trichomes on oviposition preference (Supplementary Fig. S2). For the specialist herbivore *Plutella xylostella* a negative relationship was found between trichome density and egg number on Arabidopsis plants (Handley *et al.*, 2005). In our study, glabrous accessions Est-0 and Br-0 indeed belonged to the accessions that received above median numbers of eggs per plant. However, we found no difference in oviposition preference between trichomed Col-0 and glabrous Col-5 plants, suggesting that trichomes are not an important host selection cue for *Pieris* oviposition in our experimental set-up. This might be due to the fact that *Pieris* butterflies deposit their eggs predominantly on the abaxial side of the leaf where no trichomes are present. In addition, *Pieris* butterflies are relatively large compared to *P. xylostella*, possibly explaining why they are less affected by plant trichomes. Also for *Helicoverpa zea* moths, which lay their eggs on the trichome-containing adaxial leaf side, trichome density did not seem to affect oviposition preference on tomato plants (Tian *et al.*, 2012).

### Leaf yellowing is unattractive to *Pieris* butterflies

We observed that plants with spontaneous chlorotic (i.e. yellowing) or necrotic lesions were less attractive for *Pieris* oviposition (Supplementary Table S2). Lesion-forming plants may be visually unattractive or exhibit unfavorable defenses that can be sensed by the butterflies. In black mustard, egg-induced necrosis can cause detachment of eggs from plant leaves, preventing herbivory after hatching (Shapiro and De Vay, 1987). Although we did not observe egg-induced necrosis in the 350 Arabidopsis accessions, *Pieris* butterflies apparently dislikes depositing eggs on plants displaying visual chlorotic or necrotic spots, most likely due to the unfavorable nutritional status of these plants.

### Variation in plant secondary metabolites

We also found a weak positive correlation between egg counts and indole-glucosinolate levels (Supplementary Table S2) as determined by Kliebenstein *et al.* (2001), confirming previous findings that *Pieris* is stimulated by glucosinolates for host selection and feeding (Städler *et al.*, 1995; Müller *et al.*, 2010). It also validates our experimental set-up as being capable of assessing oviposition preference of *Pieris* butterflies. While the correlation between egg counts and indole-glucosinolate levels was non-significantly positive, the correlation with aliphatic glucosinolate levels was moderate to strong and significantly negative. A possible explanation is that plants that predominantly have indole-glucosinolates stimulate oviposition by *Pieris*, thereby resulting in less oviposition on plants that predominantly have aliphatic glucosinolates (Huang and Renwick, 1994; Kliebenstein *et al.*, 2001; De Vos *et al.*, 2008; Müller *et al.*, 2010).

### Data correction and GWAS results

To limit effects unrelated to plant genotype, we randomized the position of all 350 accessions within all seven experiments and normalized the egg counts per plant for the overall cage position effects and the total number of eggs deposited (Supplementary Table S2). After normalization, we still observed differences in the number of eggs that were deposited on the accessions, which can be explained by the genetic variation among the accessions. Morphological plant traits (e.g. plant size) were not taken along as cofactors during our GWAS, as there is evidence for genetic connections between plant growth and (constitutive) defenses (Bechtold *et al.*, 2010).

The obtained GWAS associations revealed 10 candidate loci (with 11 SNP associations) of which several were previously linked to plant traits affecting herbivory, stress signaling or general defensive mechanisms (Fig. 2A; Table 1; Supplementary Table S4). Since a GWAS associates phenotypes to loci instead of causal SNPs, additional fine mapping is required to elucidate potential gene candidates.

### Fine mapping revealed *AOC1* as a candidate gene affecting oviposition preference

To identify causal SNPs, fine mapping was performed providing additional evidence for the observed associations with our GWAS (Fig. 2B; Supplementary Fig. S3;  Supplementary Table S5). Among them, we found a significant SNP–trait association with *AOC1* and oviposition preference, and we obtained supporting evidence for this in AOC-impaired Arabidopsis plants (Fig. 3; Supplementary Fig. S4). *AOC1* encodes an allene oxide cyclase that is essential for the biosynthesis of JA and its oxylipin derivatives. JA biosynthesis is known to be an essential step in induced defense against insect herbivores (De Vos *et al.*, 2006a; Little *et al.*, 2007; Verhage *et al.*, 2011; Vos *et al.*, 2013; Wasternack and Hause, 2013). Bruinsma *et al.* (2007) showed that *P. rapae* butterflies lay more eggs on control plants over JA-treated plants, suggesting that JA-levels influence oviposition preference of *P. rapae.* Furthermore, the developmental time from larval hatching until pupation was shown to be delayed on JA-treated plants, which may be an incentive for *Pieris* butterflies to avoid oviposition on plants with high JA levels. In our choice assay, oviposition was significantly reduced on *myc234* plants, which lack glucosinolates and are impaired in responsiveness to JA, confirming the involvement of JA in oviposition preference by *Pieris* (Fig. 4; Fernandez-Calvo *et al.*, 2011) Perhaps the gene variants of *AOC1* within the HapMap collection correspond to differences in the biosynthesis of secondary metabolites (e.g. glucosinolates) and volatiles, which may affect host plant choice by *Pieris* butterflies.

### Identification of a role for *WRKY42* in oviposition preference and caterpillar performance

Fine mapping revealed *WRKY42* to have the clearest association with oviposition preference (Figs 2B, 3). Mutant *wrky42* plants displayed enhanced oviposition preference over wild-type Col-0 plants (Fig. 4), confirming its GWAS-predicted role in oviposition preference. It was shown previously that the same *wrky42* mutant had delayed leaf senescence and higher chlorophyll content (Niu *et al.*, 2020), possibly explaining attractiveness for oviposition. In the same study, Niu *et al.* (2020) showed that WRKY42 directly binds to the promoters of *isochorismate synthase 1* (*ICS1*) and *respiratory burst oxidase homolog F* (*RbohF*), of which the expression is reduced in the *wrky42* mutant. Since *ICS1* is involved in SA biosynthesis and lower SA and H_2_O_2_ (i.e. reactive oxygen species) content was measured in *wrky42* plants (Niu *et al.*, 2020), this might indicate that WRKY42 interferes with crosstalk between SA- and JA-dependent defenses and as such influences oviposition preference of *Pieris* butterflies. A lower SA content in *wrky42* may also alter the SA-dependent defense response that is normally found underneath deposited *Pieris* eggs and negatively affects JA-dependent defenses (Little *et al.*, 2007; Bruessow *et al.*, 2010). A reduction in SA-mediated suppression of JA-dependent defenses in egg-receiving *wrky42* plants may explain the reduced caterpillar performance (i.e. weight gain) of *Pieris* larvae on *wrky42* plants (Fig. 5). The combined findings of enhanced oviposition preference and reduced caterpillar performance on *wrky42* plants indicates that the ‘mother knows best’ hypothesis may not fit this specific case. However, although caterpillar performance is negatively affected in a no-choice laboratory test, survival and competition with other herbivores in a natural setting may still outweigh the reduced body mass.

### *Pieris rapae* versus *P. brassicae*

Using *P. brassicae* for validating genes found for *P. rapae* preference may have influenced the preference and performance tests. Ideally, we would confirm our results with *P. rapae*, which was unfortunately not possible with persistent rearing problems that are experienced in several laboratories that maintain *P. rapae* colonies. On the other hand, *P. rapae* and *P. brassicae* are highly related species, both specialized on Brassicaceae and are likely to harbor similar adaptations to their host plants. In accordance with that, we also found similar oviposition preferences between *P. rapae* and *P. brassicae* on a subset of Arabidopsis accessions (Supplementary Fig. S5).

### Concluding remarks

Our GWAS study identified the transcription factor WRKY42 as a player in both the oviposition preference and caterpillar performance of *Pieris* butterflies. Future research will be focused on understanding the mechanism by which impairment of *WRKY42* is associated with oviposition preference, while negatively impacting caterpillar performance. Is it part of a strategy of specialist herbivores to outcompete generalist herbivores that are less adapted to specific plant secondary metabolites? In addition, more candidate genes may be identified through fine mapping when full genome sequences of more Arabidopsis accessions will become available. Knowledge on plant genetics and *Pieris* oviposition preference may be used in breeding strategies that are aimed at reducing the attractiveness of crop plants for these insect herbivores.

## Supplementary data

The following supplementary data are available at *JXB* online.

Fig. S1. Experimental set-up.

Fig. S2. Oviposition preference of *P. rapae* butterflies on trichomed Col-0 versus glabrous Col-5 Arabidopsis plants.

Fig. S3. Fine mapping results of GWAS SNP–trait associations.

Fig. S4. Amino acid changes of fine mapping candidate genes.

Fig. S5. Oviposition preference by *P. brassicae*.

Table S1. Average number of eggs deposited per plant on each position within the experimental set-up (Fig. 1).

Table S2. Average number of eggs deposited per plant for 350 Arabidopsis accessions of the HapMap collection.

Table S3. Input data for GWAS.

Table S4. Arabidopsis loci of SNP–trait associations and underlying candidate genes within 50-kb window of each SNP.

Table S5. Accessions used for fine mapping.

erac501_suppl_Supplementary_Tables

erac501_suppl_Supplementary_Figures

## Data Availability

All data supporting the findings of this study are available within the paper and within its supplementary data published online.

## Abbreviations:

AOC1: allene oxide cyclase 1
GWAS: genome-wide association study
JA: jasmonic acid
SA: salicylic acid
WRKY42: WRKY-motif transcription factor 42

## References

[CIT0001] Ali JG , Agrawal AA. 2012. Specialist versus generalist insect herbivores and plant defense. Trends in Plant Science 17, 293–302. 22425020 10.1016/j.tplants.2012.02.006

[CIT0002] Atwell S , Huang YS, Vilhjálmsson BJ, et al. 2010. Genome-wide association study of 107 phenotypes in *Arabidopsis thaliana* inbred lines. Nature 465, 627–631. 20336072 10.1038/nature08800 PMC3023908

[CIT0003] Barth S , Melchinger AE, Lubberstedt T. 2002. Genetic diversity in *Arabidopsis thaliana* L. Heynh. investigated by cleaved amplified polymorphic sequence (CAPS) and inter-simple sequence repeat (ISSR) markers. Molecular Ecology 11, 495–505. 11918784 10.1046/j.0962-1083.2002.01466.x

[CIT0004] Baxter I , Brazelton JN, Yu D, et al. 2010. A coastal cline in sodium accumulation in *Arabidopsis thaliana* is driven by natural variation of the sodium transporter AtHKT1;1. PLoS Genetics 6, e1001193. 21085628 10.1371/journal.pgen.1001193 PMC2978683

[CIT0005] Bechtold U , Lawson T, Mejia-Carranza J, Meyer RC, Brown IR, Altmann T, Ton J, Mullineaux PM. 2010. Constitutive salicylic acid defences do not compromise seed yield, drought tolerance and water productivity in the *Arabidopsis* accession C24. Plant Cell & Environment 33, 1959–1973. 10.1111/j.1365-3040.2010.02198.x 20573051

[CIT0006] Benjamini Y , Hochberg Y. 1995. Controlling the false discovery rate – a practical and powerful approach to multiple testing. Journal of the Royal Statistical Society, Series B 57, 289–300.

[CIT0007] Berendzen K , Searle I, Ravenscroft D, Koncz C, Batschauer A, Coupland G, Somssich IE, Ulker B. 2005. A rapid and versatile combined DNA/RNA extraction protocol and its application to the analysis of a novel DNA marker set polymorphic between *Arabidopsis thaliana* ecotypes Col-0 and Landsberg erecta. Plant Methods 1, 4. 16270938 10.1186/1746-4811-1-4 PMC1277017

[CIT0008] Bloomer RH , Juenger TE, Symonds VV. 2012. Natural variation in GL1 and its effects on trichome density in *Arabidopsis thaliana*. Molecular Ecology 21, 3501–3515. 22625421 10.1111/j.1365-294X.2012.05630.x

[CIT0009] Bodenhausen N , Reymond P. 2007. Signaling pathways controlling induced resistance to insect herbivores in *Arabidopsis*. Molecular Plant-Microbe Interactions 20, 1406–1420. 17977152 10.1094/MPMI-20-11-1406

[CIT0010] Brash AR , Baertschi SW, Ingram CD, Harris TM. 1988. Isolation and characterization of natural allene oxides: unstable intermediates in the metabolism of lipid hydroperoxides. Proceedings of the National Academy of Sciences, USA 85, 3382–3386. 10.1073/pnas.85.10.3382 PMC280213 2835769

[CIT0011] Broekgaarden C , Caarls L, Vos IA, Pieterse CMJ, Van Wees SCM. 2015. Ethylene: traffic controller on hormonal crossroads to defense. Plant Physiology 169, 2371–2379. 26482888 10.1104/pp.15.01020 PMC4677896

[CIT0012] Bruessow F , Gouhier-Darimont C, Buchala A, Metraux J-P, Reymond P. 2010. Insect eggs suppress plant defence against chewing herbivores. The Plant Journal 62, 876–885. 20230509 10.1111/j.1365-313X.2010.04200.x

[CIT0013] Bruinsma M , Dam NM, Van Loon JJA, Dicke M. 2007. Jasmonic acid-induced changes in *Brassica oleracea* affect oviposition preference of two specialist herbivores. Journal of Chemical Ecology 33, 655–668. 17334923 10.1007/s10886-006-9245-2 PMC1915630

[CIT0014] Bukovinszky T , Potting RPJ, Clough Y, Lenteren JC, Vet LEM. 2005. The role of pre- and post-alighting detection mechanisms in the responses to patch size by specialist herbivores. Oikos 109, 435–446.

[CIT0015] Chao D-Y , Silva A, Baxter I, Huang Y, Nordborg M, Danku J, Lahner B, Yakubova E, Salt D. 2012. Genome-wide association studies identify heavy metal ATPase3 as the primary determinant of natural variation in leaf cadmium in *Arabidopsis thaliana*. PLoS Genetics 8, e1002923. 22969436 10.1371/journal.pgen.1002923 PMC3435251

[CIT0016] Coolen S , Van Pelt JA, Van Wees SCM, Pieterse CMJ. 2019. Mining the natural genetic variation in *Arabidopsis thaliana* for adaptation to sequential abiotic and biotic stresses. Planta 249, 1087–1105. 30547240 10.1007/s00425-018-3065-9

[CIT0017] Dabrowska P , Freitak D, Vogel H, Heckel DG, Boland W. 2009. The phytohormone precursor OPDA is isomerized in the insect gut by a single, specific glutathione transferase. Proceedings of the National Academy of Sciences, USA 106, 16304–16309. 10.1073/pnas.0906942106 PMC2752597 19805297

[CIT0018] Davila Olivas NH , Frago E, Thoen MPM, Kloth KJ, Becker FFM, Van Loon JJA, Gort G, Keurentjes JJB, van Heerwaarden J, Dicke M. 2017a. Natural variation in life history strategy of *Arabidopsis thaliana* determines stress responses to drought and insects of different feeding guilds. Molecular Ecology 26, 2959–2977. 28295823 10.1111/mec.14100 PMC5485070

[CIT0019] Davila Olivas NH , Kruijer W, Gort G, Wijnen CL, Van Loon JJA, Dicke M. 2017b. Genome-wide association analysis reveals distinct genetic architectures for single and combined stress responses in *Arabidopsis thaliana*. New Phytologist 213, 838–851. 27604707 10.1111/nph.14165 PMC5217058

[CIT0020] De Vos M , Denekamp M, Dicke M, Vuylsteke M, Van Loon LC, Smeekens SCM, Pieterse CMJ. 2006a. The *Arabidopsis thaliana* transcription factor AtMYB102 functions in defense against the insect herbivore *Pieris rapae*. Plant Signaling & Behavior 1, 305–311. 19517001 10.4161/psb.1.6.3512 PMC2634245

[CIT0021] De Vos M , Kriksunov KL, Jander G. 2008. Indole-3-acetonitrile production from indole glucosinolates deters oviposition by *Pieris rapae*. Plant Physiology 146, 916–926. 18192443 10.1104/pp.107.112185 PMC2259081

[CIT0022] De Vos M , Van Zaanen W, Koornneef A, Korzelius JP, Dicke M, Van Loon LC, Pieterse CMJ. 2006b. Herbivore-induced resistance against microbial pathogens in Arabidopsis. Plant Physiology 142, 352–363. 16829584 10.1104/pp.106.083907 PMC1557608

[CIT0023] Delker C. 2005. Jasmonatbiosynthese in *Arabidopsis thaliana* – Charakterisierung der allenoxidcyclase-genfamilie und von mutanten der fettsäure-β-oxidation. Ph.D Thesis, Marthin Luther University Halle-Wittenberg, Germany.

[CIT0024] Edger PP , Heidel-Fischer HM, Bekaert M, et al. 2015. The butterfly plant arms-race escalated by gene and genome duplications. Proceedings of the National Academy of Sciences, USA 112, 8362–8366. 10.1073/pnas.1503926112 PMC4500235 26100883

[CIT0025] Erb M , Reymond P. 2019. Molecular interactions between plants and insect herbivores. Annual Review of Plant Biology 70, 527–557. 10.1146/annurev-arplant-050718-095910 30786233

[CIT0026] Fatouros NE , Cusumano A, Danchin E, Colazza S. 2016. Prospects of herbivore egg-killing plant defenses for sustainable crop protection. Ecology and Evolution 6, 6906–6918. 28725368 10.1002/ece3.2365 PMC5513223

[CIT0027] Fatouros NE , Lucas-Barbosa D, Weldegergis BT, Pashalidou FG, Van Loon JJA, Dicke M, Harvey JA, Gols R, Huigens ME. 2012. Plant volatiles induced by herbivore egg deposition affect insects of different trophic levels. PLoS One 7, e43607. 22912893 10.1371/journal.pone.0043607 PMC3422343

[CIT0028] Fernandez-Calvo P , Chini A, Fernandez-Barbero G, et al. 2011. The Arabidopsis bHLH transcription factors MYC3 and MYC4 are targets of JAZ repressors and act additively with MYC2 in the activation of jasmonate responses. The Plant Cell 23, 701–715. 21335373 10.1105/tpc.110.080788 PMC3077776

[CIT0029] Frerigmann H , Böttcher C, Baatout D, Gigolashvili T. 2012. Glucosinolates are produced in trichomes of *Arabidopsis thaliana*. Frontiers in Plant Science 3, 242. 23115560 10.3389/fpls.2012.00242 PMC3483630

[CIT0030] Griese E , Pineda A, Pashalidou FG, Iradi EP, Hilker M, Dicke M, Fatouros NE. 2020. Plant responses to butterfly oviposition partly explain preference-performance relationships on different brassicaceous species. Oecologia 192, 463–475. 31932923 10.1007/s00442-019-04590-y PMC7002336

[CIT0031] Groux R , Hilfiker O, Gouhier-Darimont C, Penaflor MF, Erb M, Reymond P. 2014. Role of methyl salicylate on oviposition deterrence in *Arabidopsis thaliana*. Journal of Chemical Ecology 40, 754–759. 24973956 10.1007/s10886-014-0470-9

[CIT0032] Handley R , Ekbom B, Ågren J. 2005. Variation in trichome density and resistance against a specialist insect herbivore in natural populations of *Arabidopsis thaliana*. Ecological Entomology 30, 284–292.

[CIT0033] Harvey JA , Witjes LMA, Benkirane M, Duyts H, Wagenaar R. 2007. Nutritional suitability and ecological relevance of *Arabidopsis thaliana* and *Brassica oleracea* as foodplants for the cabbage butterfly, *Pieris rapae*. Plant Ecology 189, 117–126.

[CIT0034] Hauser MT , Harr B, Schlotterer C. 2001. Trichome distribution in *Arabidopsis thaliana* and its close relative *Arabidopsis lyrata*: molecular analysis of the candidate gene *GLABROUS1*. Molecular Biology and Evolution 18, 1754–1763. 11504855 10.1093/oxfordjournals.molbev.a003963

[CIT0035] Hern A , Edwards-Jones G, McKinlay RG. 1996. A review of the cabbage white pre-oviposition behaviour of the small butterfly, *Pieris rapae* (Lepidoptera: Pieridae). Annals of Applied Biology 128, 349–371.

[CIT0036] Hopkins RJ , Van Dam NM, Van Loon JJA. 2009. Role of glucosinolates in insect-plant relationships and multitrophic interactions. Annual Review of Entomology 54, 57–83. 10.1146/annurev.ento.54.110807.090623 18811249

[CIT0037] Howe GA , Jander G. 2008. Plant immunity to insect herbivores. Annual Review of Plant Biology 59, 41–66. 10.1146/annurev.arplant.59.032607.092825 18031220

[CIT0038] Howe GA , Schilmiller AL. 2002. Oxylipin metabolism in response to stress. Current Opinion in Plant Biology 5, 230–236. 11960741 10.1016/s1369-5266(02)00250-9

[CIT0039] Huang X , Renwick JAA. 1994. Relative activities of glucosinolates as oviposition stimulants for *Pieris rapae* and *P. napi oleracea*. Journal of Chemical Ecology 20, 1025–1037. 24242300 10.1007/BF02059739

[CIT0040] Hwang S-Y , Liu C-H, Shen T-C. 2008. Effects of plant nutrient availability and host plant species on the performance of two *Pieris* butterflies (Lepidoptera: Pieridae). Biochemical Systematics and Ecology 36, 505–513.

[CIT0041] Johanson U , West J, Lister C, Michaels S, Amasino R, Dean C. 2000. Molecular analysis of *FRIGIDA*, a major determinant of natural variation in *Arabidopsis* flowering time. Science 290, 344–347. 11030654 10.1126/science.290.5490.344

[CIT0042] Jones RE. 1977. Movement patterns and egg distribution in cabbage butterflies. Journal of Animal Ecology 46, 195–212.

[CIT0043] Karssemeijer PN , Winzen L, Van Loon JJA, Dicke M. 2022. Leaf-chewing herbivores affect preference and performance of a specialist root herbivore. Oecologia 199, 243–255. 35192063 10.1007/s00442-022-05132-9 PMC9226102

[CIT0044] Kessler A , Halitschke R, Baldwin IT. 2004. Silencing the jasmonate cascade: induced plant defenses and insect populations. Science 305, 665–668. 15232071 10.1126/science.1096931

[CIT0045] Kim S , Plagnol V, Hu TT, Toomajian C, Clark RM, Ossowski S, Ecker JR, Weigel D, Nordborg M. 2007. Recombination and linkage disequilibrium in *Arabidopsis thaliana*. Nature Genetics 39, 1151–1155. 17676040 10.1038/ng2115

[CIT0046] Kliebenstein DJ , Kroymann J, Brown P, Figuth A, Pedersen D, Gershenzon J, Mitchell-Olds T. 2001. Genetic control of natural variation in Arabidopsis glucosinolate accumulation. Plant Physiology 126, 811–825. 11402209 10.1104/pp.126.2.811 PMC111171

[CIT0047] Kloth KJ , Wiegers GL, Busscher-Lange J, van Haarst JC, Kruijer W, Bouwmeester HJ, Dicke M, Jongsma MA. 2016. AtWRKY22 promotes susceptibility to aphids and modulates salicylic acid and jasmonic acid signalling. Journal of Experimental Botany 67, 3383–3396. 27107291 10.1093/jxb/erw159 PMC4892728

[CIT0048] Koo AJ. 2017. Metabolism of the plant hormone jasmonate: a sentinel for tissue damage and master regulator of stress response. Phytochemistry Reviews 17, 51–80.

[CIT0049] Koornneef M , Alonso-Blanco C, Vreugdenhil D. 2004. Naturally occurring genetic variation in *Arabidopsis thaliana*. Annual Review of Plant Biology 55, 141–172. 10.1146/annurev.arplant.55.031903.141605 15377217

[CIT0050] Kruijer W , Boer MP, Malosetti M, Flood PJJ, Engel B, Kooke R, Keurentjes JJ, Van Eeuwijk FA. 2015. Marker-based estimation of heritability in immortal populations. Genetics 199, 379–398. 25527288 10.1534/genetics.114.167916 PMC4317649

[CIT0051] Leon-Reyes A , der Does D, Lange ES, Delker C, Wasternack C, Wees SCM, Ritsema T, Pieterse CMJ. 2010. Salicylate-mediated suppression of jasmonate-responsive gene expression in Arabidopsis is targeted downstream of the jasmonate biosynthesis pathway. Planta 232, 1423–1432. 20839007 10.1007/s00425-010-1265-z PMC2957573

[CIT0052] Lippert C , Listgarten J, Liu Y, Kadie CM, Davidson RI, Heckerman D. 2011. FaST linear mixed models for genome-wide association studies. Nature Methods 8, 833–835. 21892150 10.1038/nmeth.1681

[CIT0053] Little D , Gouhier-Darimont C, Bruessow F, Reymond P. 2007. Oviposition by pierid butterflies triggers defense responses in Arabidopsis. Plant Physiology 143, 784–800. 17142483 10.1104/pp.106.090837 PMC1803735

[CIT0054] Lortzing V , Oberlander J, Lortzing T, Tohge T, Steppuhn A, Kunze R, Hilker M. 2019. Insect egg deposition renders plant defence against hatching larvae more effective in a salicylic acid-dependent manner. Plant, Cell & Environment 42, 1019–1032. 10.1111/pce.13447 30252928

[CIT0055] Müller R , De Vos M, Sun JY, Sønderby IE, Halkier BA, Wittstock U, Jander G. 2010. Differential effects of indole and aliphatic glucosinolates on Lepidopteran herbivores. Journal of Chemical Ecology 36, 905–913. 20617455 10.1007/s10886-010-9825-z

[CIT0056] Mumm R , Burow M, Bukovinszkine’Kiss G, Kazantzidou E, Wittstock U, Dicke M, Gershenzon J. 2008. Formation of simple nitriles upon glucosinolate hydrolysis affects direct and indirect defense against the specialist herbivore, *Pieris rapae*. Journal of Chemical Ecology 34, 1311–1321. 18787901 10.1007/s10886-008-9534-z

[CIT0057] Muriel SB , Grez AA. 2002. Effect of plant patch shape on the distribution and abundance of three lepidopteran species associated with *Brassica oleracea*. Agricultural and Forest Entomology 4, 179–185.

[CIT0058] Myers JH. 1985. Effect of physiological condition of the host plant on the ovipositional choice of the cabbage white butterfly, *Pieris rapae*. Journal of Animal Ecology 54, 193–204.

[CIT0059] Niu F , Cui X, Zhao P, Sun M, Yang B, Deyholos MK, Li Y, Zhao X, Jiang YQ. 2020. WRKY42 transcription factor positively regulates leaf senescence through modulating SA and ROS synthesis in *Arabidopsis thaliana*. The Plant Journal 104, 171–184. 32634860 10.1111/tpj.14914

[CIT0060] Nordborg M , Hu TT, Ishino Y, et al. 2005. The pattern of polymorphism in *Arabidopsis thaliana*. PLoS Biology 3, e196. 15907155 10.1371/journal.pbio.0030196 PMC1135296

[CIT0061] Okamura Y , Sato A, Tsuzuki N, Sawada Y, Hirai MY, Heidel-Fischer H, Reichelt M, Murakami M, Vogel H. 2019. Differential regulation of host plant adaptive genes in *Pieris* butterflies exposed to a range of glucosinolate profiles in their host plants. Scientific Report 9, 7256. 10.1038/s41598-019-43703-8 PMC6510735 31076616

[CIT0062] Pangesti N , Reichelt M, van de Mortel JE, Kapsomenou E, Gershenzon J, van Loon JJ, Dicke M, Pineda A. 2016. Jasmonic acid and ethylene signaling pathways regulate glucosinolate levels in plants during rhizobacteria-induced systemic resistance against a leaf-chewing herbivore. Journal of Chemical Ecology 42, 1212–1225. 27848154 10.1007/s10886-016-0787-7 PMC5148788

[CIT0063] Pieterse CMJ , Dicke M. 2007. Plant interactions with microbes and insects: from molecular mechanisms to ecology. Trends in Plant Science 12, 564–569. 17997347 10.1016/j.tplants.2007.09.004

[CIT0064] Pigliucci M. 1998. Ecological and evolutionary genetics of *Arabidopsis*. Trends in Plant Science 3, 485–489.

[CIT0065] Platt A , Horton M, Huang YS, et al. 2010. The scale of population structure in *Arabidopsis thaliana*. PLoS Genetics 6, e1000843. 20169178 10.1371/journal.pgen.1000843 PMC2820523

[CIT0066] Proietti S , Caarls L, Coolen S, Van Pelt JA, Van Wees SCM, Pieterse CMJ. 2018. Genome-wide association study reveals novel players in defense hormone crosstalk in *Arabidopsis*. Plant Cell & Environment 41, 2342–2356. 10.1111/pce.13357 PMC6175328 29852537

[CIT0067] Renwick JAA , Radke CD. 1988. Sensory cues in host selection for oviposition by the cabbage butterfly, *Pieris rapae*. Journal of Insect Physiology 34, 251–257.

[CIT0068] Reymond P , Bodenhausen N, Dicke M, Farmer EE. 2004. A conserved transcript pattern in response to a specialist and a generalist herbivore. The Plant Cell 16, 3132–3147. 15494554 10.1105/tpc.104.026120 PMC527203

[CIT0069] Root RB. 1973. Organization of a plant-arthropod association in simple and diverse habitats: the fauna of collards (*Brassica oleracea*). Ecological Monographs 43, 95–124.

[CIT0070] Root RB , Kareiva PM. 1984. The search for resources by cabbage butterflies (*Pieris rapae*): ecological consequences and adaptive significance of markovian movements in a patchy environment. Ecology 65, 147–165.

[CIT0071] Schoonhoven LM , Van Loon JJA, Dicke M. 2005. Insect-plant biology. Oxford: Oxford University Press.

[CIT0072] Schweizer F , Fernandez-Calvo P, Zander M, Diez-Diaz M, Fonseca S, Glauser G, Lewsey MG, Ecker JR, Solano R, Reymond P. 2013. *Arabidopsis* basic helix-loop-helix transcription factors MYC2, MYC3, and MYC4 regulate glucosinolate biosynthesis, insect performance, and feeding behavior. The Plant Cell 25, 3117–3132. 23943862 10.1105/tpc.113.115139 PMC3784603

[CIT0073] Shabab M , Khan SA, Vogel H, Heckel DG, Boland W. 2014. OPDA isomerase GST16 is involved in phytohormone detoxification and insect development. FEBS Journal 281, 2769–2783. 24730650 10.1111/febs.12819

[CIT0074] Shapiro AM , De Vay JE. 1987. Hypersensitivity reaction of *Brassica nigra* L. (Cruciferae) kills eggs of *Pieris* butterflies (Lepidoptera: Pieridae). Oecologia 71, 631–632. 28312240 10.1007/BF00379310

[CIT0075] Smallegange RC , Everaarts TC, Van Loon JJA. 2006. Associative learning of visual and gustatory cues in the large cabbage white butterfly, *Pieris brassicae*. Animal Biology 56, 157–172.

[CIT0076] Snoeren TA , Van Poecke RM, Dicke M. 2009. Multidisciplinary approach to unravelling the relative contribution of different oxylipins in indirect defense of *Arabidopsis thaliana*. Journal of Chemical Ecology 35, 1021–1031. 19798534 10.1007/s10886-009-9696-3 PMC2759439

[CIT0077] Soler R , Erb M, Kaplan I. 2013. Long distance root-shoot signalling in plant-insect community interactions. Trends in Plant Science 18, 149–156. 22989699 10.1016/j.tplants.2012.08.010

[CIT0078] Song WC , Brash AR. 1991. Purification of an allene oxide synthase and identification of the enzyme as a cytochrome P-450. Science 253, 781–784. 1876834 10.1126/science.1876834

[CIT0079] Städler E , Renwick JAA, Radke CD, Sachdev-Gupta K. 1995. Tarsal contact chemoreceptor response to glucosinolates and cardenolides mediating oviposition in *Pieris rape*. Physiological Entomology 20, 175–187.

[CIT0080] Stahl E , Brillatz T, Ferreira Queiroz E, Marcourt L, Schmiesing A, Hilfiker O, Riezman I, Riezman H, Wolfender JL, Reymond P. 2020. Phosphatidylcholines from *Pieris brassicae* eggs activate an immune response in Arabidopsis. eLife 9, e60293. 32985977 10.7554/eLife.60293 PMC7521926

[CIT0081] Stenzel I , Otto M, Delker C, Kirmse N, Schmidt D, Miersch O, Hause B, Wasternack C. 2012. *ALLENE OXIDE CYCLASE* (*AOC*) gene family members of *Arabidopsis thaliana*: tissue- and organ-specific promoter activities and *in vivo* heteromerization. Journal of Experimental Botany 63, 6125–6138. 23028017 10.1093/jxb/ers261 PMC3481204

[CIT0082] Su T , Xu Q, Zhang FC, Chen Y, Li LQ, Wu WH, Chen YF. 2015. WRKY42 modulates phosphate homeostasis through regulating phosphate translocation and acquisition in Arabidopsis. Plant Physiology 167, 1579–1591. 25733771 10.1104/pp.114.253799 PMC4378159

[CIT0083] Thoen MPM , Davila Olivas NH, Kloth KJ, et al. 2017. Genetic architecture of plant stress resistance: multi-trait genome-wide association mapping. New Phytologist 213, 1346–1362. 27699793 10.1111/nph.14220 PMC5248600

[CIT0084] Tian D , Tooker J, Peiffer M, Chung SH, Felton GW. 2012. Role of trichomes in defense against herbivores: comparison of herbivore response to woolly and hairless trichome mutants in tomato (*Solanum lycopersicum*). Planta 236, 1053–1066. 22552638 10.1007/s00425-012-1651-9

[CIT0085] Todesco M , Balasubramanian S, Hu TT, et al. 2010. Natural allelic variation underlying a major fitness trade-off in *Arabidopsis thaliana*. Nature 465, 632–636. 20520716 10.1038/nature09083 PMC3055268

[CIT0086] Traynier RMM , Truscott RJW. 1991. Potent natural egg-laying stimulant for cabbage butterfly *Pieris rapae*. Journal of Chemical Ecology 17, 1371–1380. 24257798 10.1007/BF00983770

[CIT0087] Van Loon JJA , Blaakmeer A, Griepink FC, Van Beek TA, Schoonhoven LM, De Groot A. 1992. Leaf surface compound from *Brassica oleracea* (Cruciferae) induces oviposition by *Pieris brassicae* (Lepidoptera: Pieridae). Chemoecology 3, 39–44.

[CIT0088] Van Wees SCM , Van Pelt JA, Bakker PAHM, Pieterse CMJ. 2013. Bioassays for assessing jasmonate-dependent defenses triggered by pathogens, herbivorous insects, or beneficial rhizobacteria. Methods in Molecular Biology 1011, 35–49. 23615986 10.1007/978-1-62703-414-2_4

[CIT0089] Verhage A , Van Wees SCM, Pieterse CMJ. 2010. Plant immunity: it’s the hormones talking, but what do they say? Plant Physiology 154, 536–540. 20921180 10.1104/pp.110.161570 PMC2949039

[CIT0090] Verhage A , Vlaardingerbroek I, Raaymakers C, Van Dam NM, Dicke M, Van Wees SCM, Pieterse CMJ. 2011. Rewiring of the jasmonate signaling pathway in Arabidopsis during insect herbivory. Frontiers in Plant Science 2, 47. 22645537 10.3389/fpls.2011.00047 PMC3355780

[CIT0091] Von Malek B , Van der Graaff E, Schneitz K, Keller B. 2002. The Arabidopsis male-sterile mutant *dde2-2* is defective in the *ALLENE OXIDE SYNTHASE* gene encoding one of the key enzymes of the jasmonic acid biosynthesis pathway. Planta 216, 187–192. 12430030 10.1007/s00425-002-0906-2

[CIT0092] Vos IA , Moritz L, Pieterse CMJ, Van Wees SCM. 2015. Impact of hormonal crosstalk on plant resistance and fitness under multi-attacker conditions. Frontiers in Plant Science 6, 639. 26347758 10.3389/fpls.2015.00639 PMC4538242

[CIT0093] Vos IA , Verhage A, Schuurink RC, Watt LG, Pieterse CMJ, Van Wees SCM. 2013. Onset of herbivore-induced resistance in systemic tissue primed for jasmonate-dependent defenses is activated by abscisic acid. Frontiers in Plant Science 4, 539. 24416038 10.3389/fpls.2013.00539 PMC3874679

[CIT0094] Wasternack C , Hause B. 2013. Jasmonates: biosynthesis, perception, signal transduction and action in plant stress response, growth and development. An update to the 2007 review in *Annals of Botany*. Annals of Botany 111, 1021–1058. 23558912 10.1093/aob/mct067 PMC3662512

[CIT0095] Waterhouse AM , Procter JB, Martin DMA, Clamp M, Barton GJ. 2009. Jalview Version 2—a multiple sequence alignment editor and analysis workbench. Bioinformatics 25, 1189–1191. 19151095 10.1093/bioinformatics/btp033 PMC2672624

[CIT0096] Weigel D , Mott R. 2009. The 1001 genomes project for *Arabidopsis thaliana*. Genome Biology 10, 107. 19519932 10.1186/gb-2009-10-5-107 PMC2718507

[CIT0097] Wittstock U , Agerbirk N, Stauber EJ, Olsen C, Hippler M, Mitchell-Olds T, Gershenzon J, Vogel H. 2004. Successful herbivore attack due to metabolic diversion of a plant chemical defense. Proceedings of the National Academy of Sciences, USA 101, 4859–4864. 10.1073/pnas.0308007101 PMC387339 15051878

[CIT0098] Zhang B , Xie D, Jin Z. 2012. Global analysis of non-coding small RNAs in *Arabidopsis* in response to jasmonate treatment by deep sequencing technology. Journal of Integrative Plant Biology 54, 73–86. 22221297 10.1111/j.1744-7909.2012.01098.x

[CIT0099] Zhang C , Lei Y, Lu C, Wang L, Wu J. 2020. MYC2, MYC3, and MYC4 function additively in wounding-induced jasmonic acid biosynthesis and catabolism. Journal of Integrative Plant Biology 62, 1159–1175. 31876387 10.1111/jipb.12902

[CIT0100] Zhao K , Aranzana MJ, Kim S, et al. 2007. An *Arabidopsis* example of association mapping in structured samples. PLoS Genetics 3, e4. 17238287 10.1371/journal.pgen.0030004 PMC1779303

[CIT0101] Zheng S-J , Snoeren TAL, Hogewoning SW, Van Loon JJA, Dicke M. 2010. Disruption of plant carotenoid biosynthesis through virus-induced gene silencing affects oviposition behaviour of the butterfly *Pieris rapae*. New Phytologist 186, 733–745. 20298487 10.1111/j.1469-8137.2010.03213.x

