# Supplementary material for: Genome-wide association study reveals WRKY42 as a novel plant transcription factor that influences oviposition preference of Pieris butterflies
Source: J Exp Bot. 2022 Dec 23;74(5):1690–704. doi: 10.1093/jxb/erac501 (PMC10010613; doi:10.1093/jxb/erac501)
Supplement: erac501_suppl_Supplementary_Figures [file erac501_suppl_supplementary_figures.pdf]

Supplemental Figures

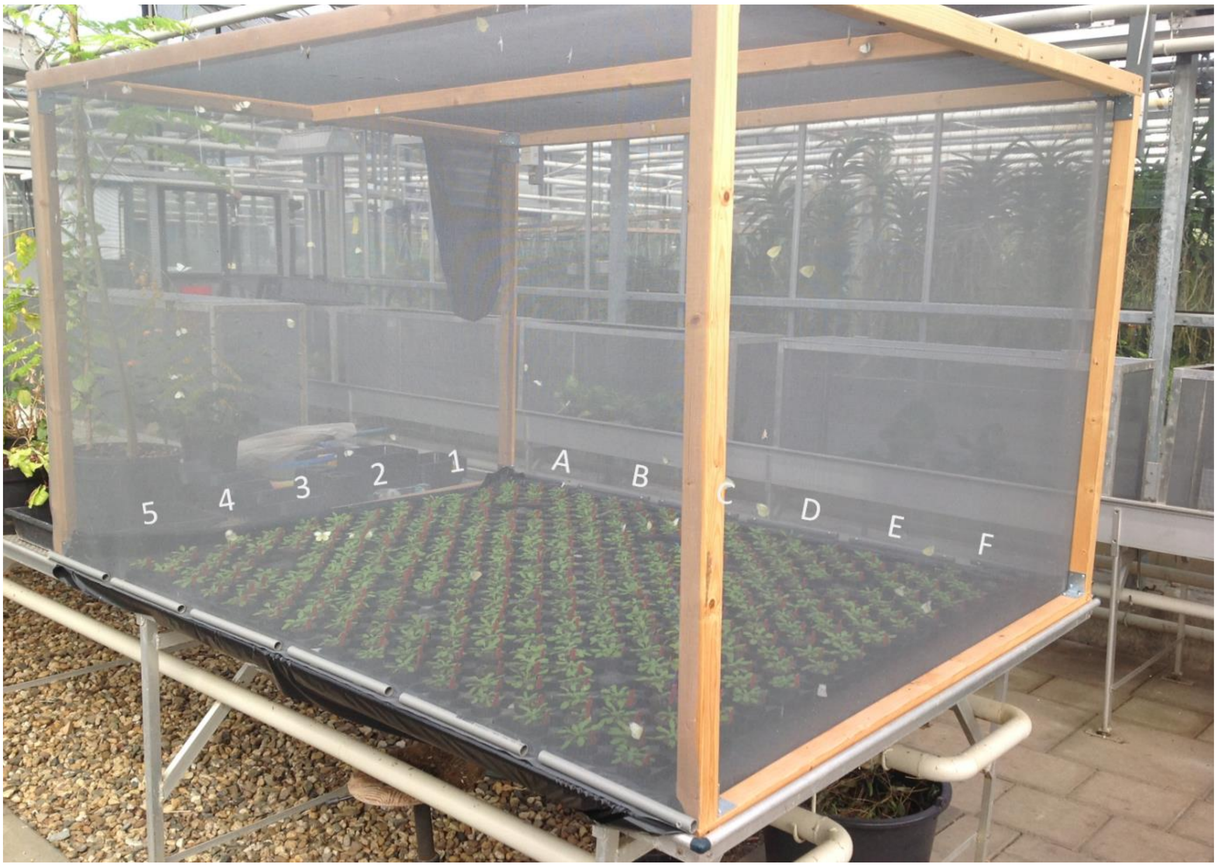

Figure S1. Experimental setup. All 350 *A. thaliana* accessions were randomly assigned a position in one of the 30 plots in the cage, after which a mixed group of 20-30 female and male butterflies were placed in the cage. Accessions were evenly spaced throughout the cage and randomized within 7 replicated experiments to correct for cage position effects. Butterflies were allowed to feed on feeding solutions supplied within the cage. Female butterflies were allowed to oviposit freely for 2-3 days.

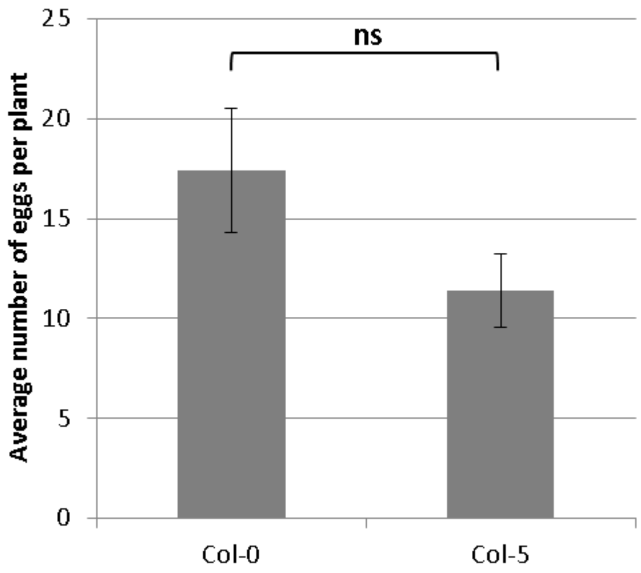

Figure S2. Oviposition preference of *P. rapae* butterflies on trichomed Col-0 versus glabrous Col-5 *A. thaliana* plants. In a setup with 5 cages, each containing 2 trichome-containing Col-0 and 2 glabrous Col-5 plants, 2 female butterflies were allowed to oviposit for 3 days. Bars represent the average number of eggs deposited per plant (n=10). Error bars represent the standard error (±SE). Significance was calculated with a Student's *t*-test ($p > 0.05$ = non-significant, ns).

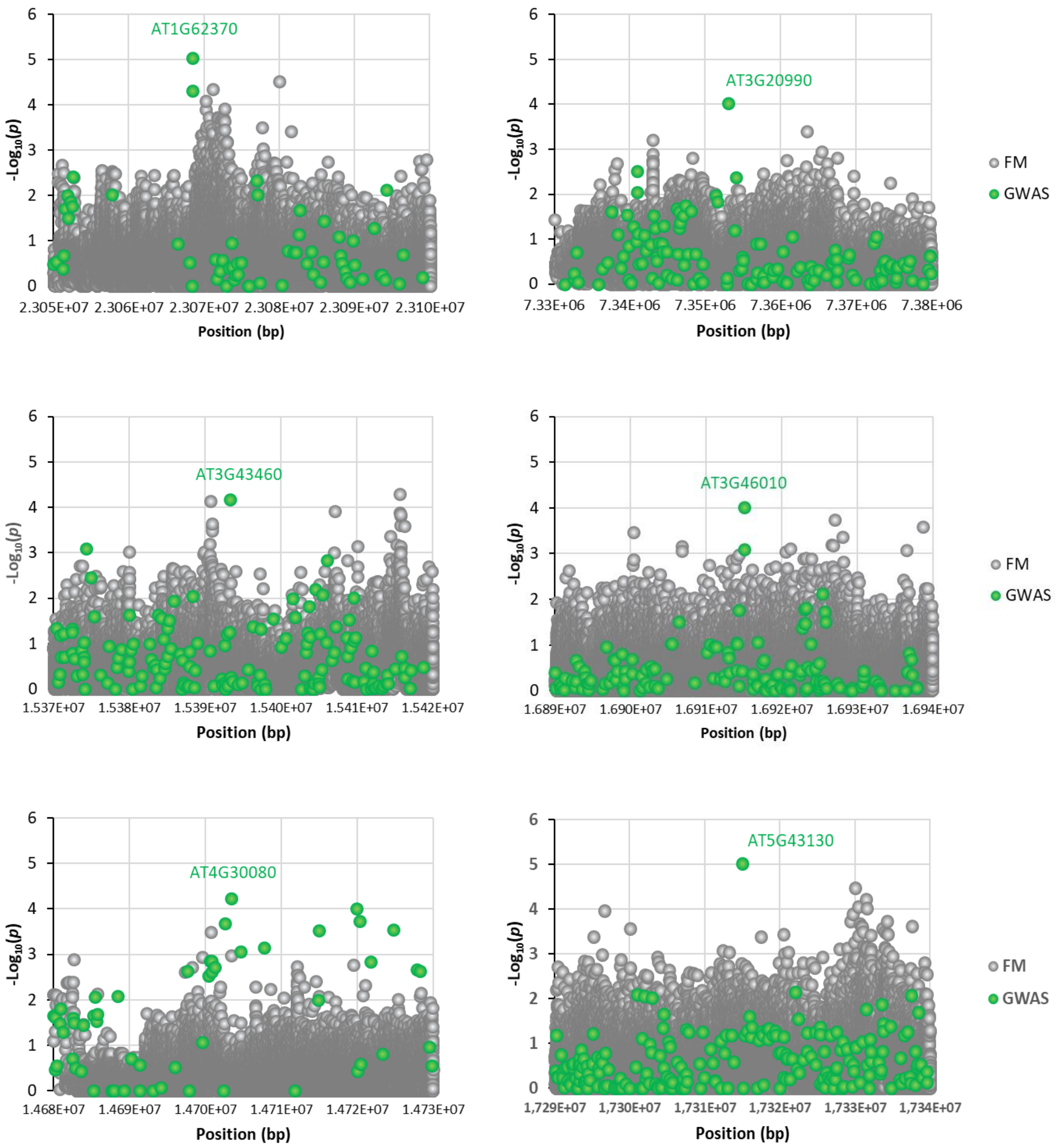

Figure S3. Fine mapping results of GWAS SNP-trait associations. Manhattan plots showing the –log10(*p*) values of the SNP-trait associations for the fine mapping results (grey dots) of 6 of the 10 loci that with GWAS (green dots) were associated with the oviposition preference of *P. rapae* (normalized average number of egg depositions per plant). On the x-axis the position in base pairs (bp) is shown. Genes indicated in green are those obtained from the GWAS SNP-trait associations.

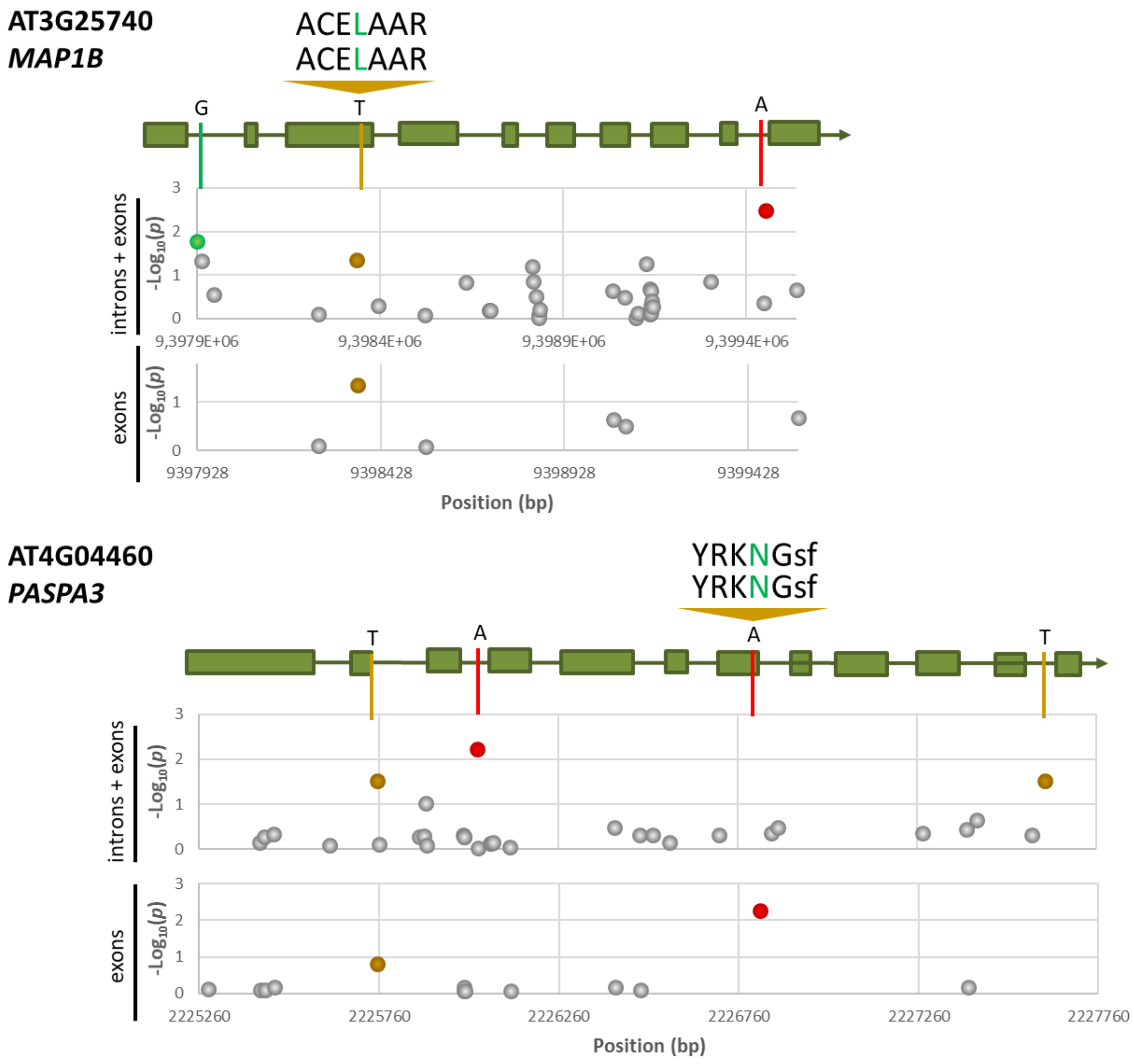

Figure S4. Amino-acid changes of fine mapping candidate genes. Manhattan plots of fine mapping results (MAF>5%) for the candidate genes AT3G25740 and AT4G04460, including both introns and exons or only exons. Y-axes show the -Log$_{10}$(*p*) and the x-axes the position in base pairs (bp). For each gene a model of the introns and exons is shown according to the 1001 genomes browser (http://signal.salk.edu/atg1001/3.0/gebrowser.php). Important (significant) nucleotides are indicated with colors: A (red), T (ochre/yellow), C (blue) and G (green). Synonymous amino acid changes are indicated with a green letter.

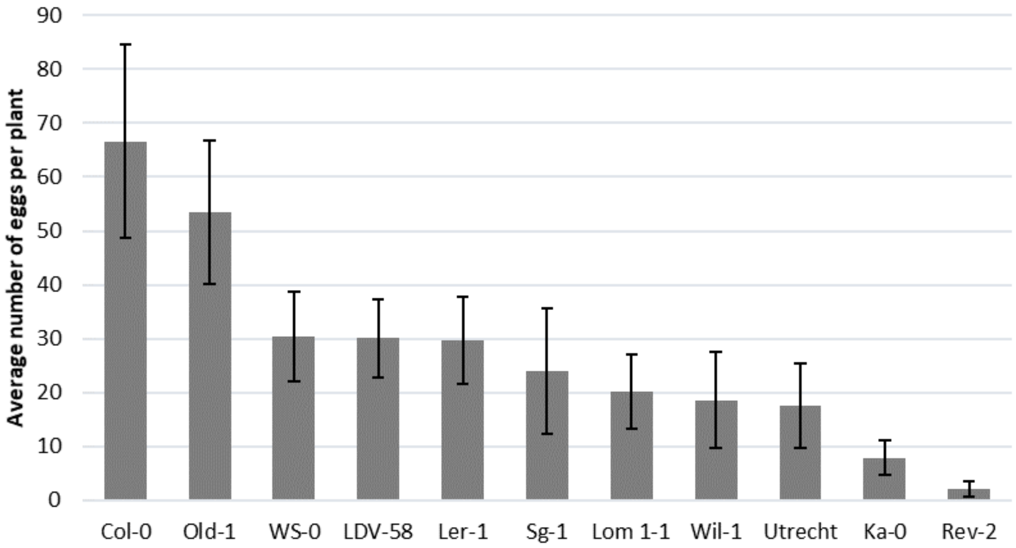

Figure S5. Oviposition preference by *P. brassicae*. The graph shows the normalized average number of eggs deposited by *P. brassicae* butterflies on 11 different *A. thaliana* accessions from the HapMap collection. Data are the average of 17 independent small cage experiments on the set of 11 accessions, each experiment containing 11 randomly positioned accession accessions. In each experiment, 1-2 female *P. rapae* butterflies were allowed to freely oviposit for 2-3 days on the offered population of 11 plants. Error bars show standard error (±SE) of mean.

Table S1. Average number of eggs deposited per plant on each position within the experimental setup (Figure 1)

Table S2. Average number of eggs deposited per plant for 350 *A. thaliana* accessions of the HapMap collection

Table S3. Input data for GWAS

Table S4. *A. thaliana* loci of SNP-trait associations and underlying candidate genes within 50-kb window of each SNP

Table S5. Accessions used for fine mapping
